# Genome-Wide Association Mapping Identifies Novel Loci for Quantitative Resistance to Blackleg Disease in Canola

**DOI:** 10.3389/fpls.2020.01184

**Published:** 2020-08-11

**Authors:** Harsh Raman, Brett McVittie, Ramethaa Pirathiban, Rosy Raman, Yuanyuan Zhang, Denise M. Barbulescu, Yu Qiu, Shengyi Liu, Brian Cullis

**Affiliations:** ^1^ NSW Department of Primary Industries, Wagga Wagga Agricultural Institute, Wagga Wagga, NSW, Australia; ^2^ Centre for Bioinformatics and Biometrics, National Institute for Applied Statistics Research Australia, University of Wollongong, Wollongong, NSW, Australia; ^3^ Oil Crops Research Institute, Chinese Academy of Agricultural Sciences, Wuhan, China; ^4^ Department of Jobs, Precincts and Regions, Agriculture Victoria, Horsham, VIC, Australia

**Keywords:** natural variation, resistance to blackleg, *Leptosphaeria maculans*, canola, genome-wide association analysis, linkage disequilibrium

## Abstract

Blackleg disease, caused by the fungal pathogen *Leptosphaeria maculans*, continues to be a major concern for sustainable production of canola (*Brassica napus* L.) in many parts of the world. The deployment of effective quantitative resistance (QR) is recognized as a durable strategy in providing natural defense to pathogens. Herein, we uncover loci for resistance to blackleg in a genetically diverse panel of canola accessions by exploiting historic recombination events which occurred during domestication and selective breeding by genome-wide association analysis (GWAS). We found extensive variation in resistance to blackleg at the adult plant stage, including for upper canopy infection. Using the linkage disequilibrium and genetic relationship estimates from 12,414 high quality SNPs, GWAS identified 59 statistically significant and “suggestive” SNPs on 17 chromosomes of *B. napus* genome that underlie variation in resistance to blackleg, evaluated under field and shade-house conditions. Each of the SNP association accounted for up to 25.1% of additive genetic variance in resistance among diverse panel of accessions. To understand the homology of QR genomic regions with *Arabidopsis thaliana* genome, we searched the synteny between QR regions with 22 ancestral blocks of Brassicaceae. Comparative analyses revealed that 25 SNP associations for QR were localized in nine ancestral blocks, as a result of genomic rearrangements. We further showed that phenological traits such as flowering time, plant height, and maturity confound the genetic variation in resistance. Altogether, these findings provided new insights on the complex genetic control of the blackleg resistance and further expanded our understanding of its genetic architecture.

## Introduction

Canola, a polyploid crop (*Brassica napus* L, 2n = 4× = 36, genome A_n_A_n_C_n_C_n_) that was only domesticated about 500 years ago (Gómez-Campo and Prakash, 1999), has now taken central stage for meeting global demands of healthy vegetable oil for human consumption. Currently, it is grown on over 37 M ha worldwide with production of 75 m tonnes (http://www.fao.org/faostat/). Blackleg, caused by the fungal pathogen *Leptosphaeria maculans*, is one of the most serious diseases of canola in many parts of the world and accounts for significant yield losses (Sosnowski et al., 2004; Fitt et al., 2006). The infection occurs at various stages of plant development and causes lesions on underground and the above ground parts; cotyledon, leaf, stem, branches, flowers, and siliques (Sprague et al., 2018). Current disease management strategies include deployment of host resistance, mediated by qualitative (race-specific *R* genes) and uncharacterized quantitative resistance (race nonspecific QTL, QR) genes, crop rotations, and application of fungicides.

Several sources of qualitative and quantitative resistance to blackleg has been identified in *Brassica* species (Mithen et al., 1987; Chevre et al., 1996; Chèvre et al., 1997; Christianson et al., 2006; Rimmer, 2006; Leflon et al., 2007; Raman et al., 2013; Fredua-Agyeman et al., 2014; Gaebelein et al., 2019); and several of them were deployed by canola breeders to develop resistant cultivars. Genetics underlying both types of resistance has been investigated in different structured mapping populations and unstructured diverse panels of canola (Rimmer, 2006; Delourme et al., 2011; Raman et al., 2013). However, loci associated with *R* gene mediated resistance are understood in greater detail compared to QR (Larkan et al., 2013; Larkan et al., 2014; Larkan et al., 2019). Though, incorporation of the *R* genes has been effective in mitigating risks for yield loss due to blackleg; many of them have become ineffective over-time, when deployed singly or in “stack” (Rouxel et al., 2003; Li et al., 2006; Sprague et al., 2006; Van De Wouw et al., 2014). In contrast, QR is recognized for its durability in providing natural defense to pathogens, including *L. maculans*, as it conveys incomplete resistance, probably due to decreased selective pressure to overcome host resistance (Johnson, 1984; Boyd, 2006; Fukuoka et al., 2009; Delourme et al., 2014).

Several hundred quantitative trait loci (QTL) for QR were identified in mapping populations following classical QTL (Delourme et al., 2006; Kaur et al., 2009; Jestin et al., 2011; Raman et al., 2012a; Raman et al., 2012b; Fopa Fomeju et al., 2014; Jestin et al., 2015; Larkan et al., 2016a; Raman et al., 2016; Kumar et al., 2018; Raman et al., 2018; Raman et al., 2020) and genome-wide association (GWAS) approaches in canola (Jestin et al., 2011; Fopa Fomeju et al., 2014; Fopa Fomeju et al., 2015; Rahman et al., 2016; Raman et al., 2016; Kumar et al., 2018). However, only a limited number of QTL provided stable QR to blackleg across environments (Pilet et al., 2001; Huang et al., 2016; Larkan et al., 2016a; Raman et al., 2018). Combining QR loci with complementary mode of action and combining QR with *R* genes could provide effective and durable resistance (Brun et al., 2010; Michelmore et al., 2017; Pilet-Nayel et al., 2017; Rimbaud et al., 2018). The latter approach has not been extremely successful in some parts of Australia, particularly when pathogen pressure is too high under field conditions (Raman et al., 2012b; Raman et al., 2020). Sources of effective QR resistance and underlying mode of action for QR genes is largely unknown in canola. Despite of immense interest in exploiting QR in canola breeding programs, comprehensive GWAS studies using larger sets of diverse panel and markers, and field-based assessments of resistance to blackleg across multiple locations and environments were lacking. In addition, upper canopy infection (UCI); characterized by lesions on siliques, pedicel, branches, and stem, at terminal stages of plant development, has become another concern for Australian canola industry in recent years (Sprague et al., 2018). Currently, there is a very limited knowledge on the extent of genetic variation for resistance to UCI in canola germplasm as well as underlying loci involved in resistance. Delineation of loci involved in multifaceted components of resistance and their uniqueness/redundancy in canola germplasm would provide potential genetic solution to reduce yield losses, caused by blackleg disease.

Herein, we assess a diverse panel of canola accessions across multiple environments and reveal significant marker-trait associations for resistance, evaluated as plant survival/mortality, internal infection (stem/crown canker) and severity of infection in upper canopy. To understand whether duplicated homoeologous regions are involved in QR, we searched the synteny between QR regions with 22 ancestral blocks (AK) of Brassicaceae (Schranz et al., 2006; Delourme et al., 2013; Fopa Fomeju et al., 2014). This analysis revealed that majority of QR regions have originated as a result of homoeologous and paralogous exchanges. We further showed that phenological traits such as flowering time, plant height and maturity confound the genetic variation for resistance. Our results provide new insights into the genetic and developmental basis for variation in QR among diverse panel of accessions. Identification of genetic variation for resistance to stem canker and UCI, in combination with molecular tools would enable the incorporation of QR into the elite germplasm, to develop new cultivars with overall reduced susceptibility to blackleg infection in canola.

## Material and Methods

### Diversity Panel

A diverse panel of 421 accessions, comprising 395 of *Brassica napus*, 21 of *B. napus*/*Brassica juncea* derivatives, one of *B. juncea* and four *of Brassica carinata* was used in this study (**Supplementary Table 1**). The *B. napus* panel includes 368 homozygous doubled haploid (DH) diverse accessions, representing different geographical locations, that were utilized earlier for mapping loci associated with photoperiodic response and flowering time (Raman et al., 2019). The DH accessions were generated *via* the microspore culture technique at the Haplotech Inc. (Manitoba, Canada).

### Phenotypic Evaluation of Diverse DH Accessions for Resistance

The diverse panel of accessions was evaluated for resistance to *L. maculans* in five experiments. Two experiments were conducted under semi-controlled shade-house conditions (SH16, SH17) across two years (2016 and 2017) at Grains Innovation Park, Horsham, Victoria, Australia (36°43’14.6”S 142°10’24.5”E) and three experiments (FT17, FT18 and FT19) were conducted under field conditions across three years (2017, 2018 and 2019) at Wagga Wagga Agricultural Institute (WWAI), Wagga Wagga, NSW, Australia (35°02′27.0″S 147°19′12.6″E).

#### Phenotyping for Resistance With Ascospore Shower Test (Shade-House Experiments)

##### Experimental Design

The shade-house experiments included a total of 327 DH accessions in two runs: in SH16, 244 accessions together with 10 current commercial cultivars (as “check”) were evaluated whereas in SH17, the remaining 77 accessions along with the same 10 “check” cultivars were evaluated (**Supplementary Table 1**). Connectivity between both experiments with 10 check cultivars and four DH accessions (46C04, ATR-Beacon, ATR-Signal and ATR-Hyden) that were used in SH16 experiment ensured that the assessment of disease expression is consistent across both experiments (**Supplementary Table 2**). In the SH16 experiment, the accessions were arranged in a Completely Randomized Design with three pot replicates for each accession (**Supplementary Figure 1A**). A total of 762 pots were located in three adjacent bays. Each bay consisted of a rectangular array of pots; each bay had either four or two columns (herein referred to as ranges) with a varying number of rows (80, 79, 32, and 31, respectively). Individual pots were the experimental units where, each pot contained 4 plants of the same accession. In the SH17 experiment, accessions were allocated to pots within three (complete) blocks. These blocks were located within one rectangular array of pots (Bay) with four ranges and 66 rows. The whole experiment had a total of 264 pots (**Supplementary Figure 1B**).

Ascospores, released from a mixed stubble were used as inoculum for the “ascospore shower” test (Huang et al., 2006b). Stubble was collected from 2015 commercial crops of Australian canola cultivars: ATR-Gem (group A: *Rlm1*, Cudal, NSW), CB-Telfer (group B: *Rlm4*, Arthurton, South Australia), ATR-Stingray (group C: *Rlm3*, Minyip, Victoria), Hyola450TT (group ABD: *Rlm1*, *Rlm4*, *LepR3*, Streatham, Victoria), Hyola650TT (group ABD, Grenfell, NSW), T28156 (group F: *Rlm6*, unreleased breeding line, Parkes, NSW) and ATR-Marlin (group S: *Rlm1*, *LepR*3, Parkes, NSW). Details of the “ascospore shower” test are given in Marcroft et al. (2012). Essentially, seedlings of test accessions were grown in punnets for approximately 10 to 15 days and then four seedlings/accession were subjected to the “ascospore shower.” The seedlings were placed onto trays and were then transferred to two growth (humidity) chambers, where the relative humidity was set at 100% at 20°C, for 48 h to encourage disease progression. After 21 days of inoculation, seedlings were transplanted into plastic pots (20 cm diameter) in a shade-house, each pot containing 4 plants per accession and subsequently raised according to the described experimental designs until the physiological maturity stage (GS 83-85). Plants were cut at the crowns and their cross-sections were assessed visually for disease severity as described previously (Marcroft et al., 2012).

#### Phenotypic Measurements of Diversity Panel Under Natural Field Conditions

A subset of the diverse panel, comprising 300 homozygous accessions (**Supplementary Table 1**) was evaluated for resistance in blackleg nursery containing mixed stubble of triazine tolerant (TT) cultivars (Raman et al., 2018) and Westar stubble at the experimental farm of WWAI (35°02’27.0”S 147°19’12.6”E).

##### Experimental Design

In each of the three field experiments, accessions were allocated to plots within two (complete) blocks. Plots consisted of a single row of at most 100 plants. These plants were grown in canola stubble of mixed TT cultivars and Westar as described in Raman et al. (2018). Each complete block comprised a rectangular array of plots with 30 rows by 10 columns separated by a buffer row of SturtTT (see **Supplementary Figure 1C**). The experiments were carried-out in canola growing seasons (April-November). The blackleg nursery was irrigated frequently (5-6 times, approximately 150 mm water applied) using lateral move, in addition to natural rainfall. The weather data and conditions for both experiments is presented in **Supplementary Figure 2**. Plant emergence was recorded by counting number of plants present in each plot after 45 days of sowing. Number of surviving plants were recorded thrice at growth stage (GS) 15-16 (after 5 weeks of emergence), GS 60-65 (at flowering stage) and at the GS 83-85 (physiological maturity). Plant survival was calculated as the proportion between number of plants at emergence and at maturity. At the physiological maturity, ten plants per accession were cut at the crown and visually assessed for internal stem infection on a 0 to 100 percentage scale based on area showing necrosis (Raman et al., 2012b).

Resistance for UCI severity was assessed in the two experiments conducted in 2018 and 2019. Number of plants infected and the extent of UCI on ten randomly selected plants from each plot were scored. An ordinal disease severity rating scale was used based on visual assessment; 0: immune reaction (no visible symptoms of UCI); (1) disease present on up to 25% of plant tissue (small lesions, resistant to UCI); (2) disease present on 26%–50% of plant tissue; (3) disease present on 51%–75% of plant tissue, (4) disease present on >75% but less than 90% of plant tissue and (5) 100% susceptibility, affecting all tissues, characterized with extensive discoloration, snapping-off vegetative (main and secondary branches) and reproductive structures (siliques).

### Measurement of Phenological Traits

Each field experiment was also assessed for three key developmental traits: flowering time, plant height and maturity. Flowering time (days to first flower, GS 65) was recorded, as the difference between the date when 50% of plants in a plot had the first flower opened and the day of sowing. The date of first flower was recorded for each plot three times in a week. Plant height (cm) was measured from top to base with a scale at the silique-filling stage (GS 80) from five plants selected randomly in the middle of each plot while, plant maturity (0–9 scale) was recorded, just 1–2 days before the assessment of blackleg severity.

### SNP Genotyping and Selection of Marker Panel

DNA was genotyped with Illumina Infinium SNP markers (Clarke et al., 2016), as detailed in our earlier study (Raman et al., 2019). Across all accessions and SNPs, there was a total of 2.7% missing values. Missing values were imputed using the *k* nearest neighbor method (Troyanskaya et al., 2001) using the *Pedicure* package (Butler, 2019) in *R* statistical computing environment (R Core Team, 2019). In this method, a missing value for a marker is replaced by the mean of its *k* nearest neighbors with nonmissing data for that individual. SNPs that had <80% call rate, and <2% MAF (minor allele frequency) were discarded prior to GWAS analysis. We examined redundant SNPs based on the Hamming distance between marker pairs. Sets of markers, whose Hamming distance was less than a threshold of 0.001 were considered as identical and were removed prior to analysis. We also removed SNPs which could not be anchored to the 19 linkage groups of A_n_, and C_n_ subgenomes (plus Ann_random and Cnn_random linkage groups) of reference sequenced genome of *B. napus* cv. *“*Darmor-*bzh”* version 4.1. A final set of 12,414 high quality SNPs for 344 accessions were selected for GWAS analysis.

### Statistical Analysis

The approach used herein for the determination of genomic regions that influence the expression of the traits associated with blackleg resistance is an extension of the whole genome, single step QTL analysis developed by Verbyla et al. (2007). Our approach uses an alternative working model which assumes the variance of each set of markers on different linkage groups (LGs) have different variances. That is if *α* is the *r*-vector of marker effects presented in map order, then our working model assumes that *α* is Gaussian, mean zero, variance

$$var\left(\alpha \right)=\;{⊕}_{i=1}^{c}\sigma_{\alpha_{i}}^{2}I_{r_{i}}$$

where *c* is the number of LGs, *r_i_* is the number of markers in the *i*th LG, $I_{r_{i}}$ is the identity matrix of order *r_i_*, ⊕ is the direct sum operator and $r=\;\sum_{i=1}^{c}r_{i}$. The working model of Verbyla et al. (2007) assumes that $\sigma_{\alpha_{i}}^{2}=\;\sigma_{\alpha }^{2}$, for *i* = 1,…, *c*. Hence their model is equivalent to the standard GBLUP model used in models for genomic selection (Tolhurst et al., 2019).

Construction of the appropriate genetic and nongenetic working model requires the inclusion of terms which represent the plot structure of the experiment(s), as well as accounting for other significant sources of nongenetic variation which occur in either shade-house studies or field trials (Gilmour et al., 1997; Smith et al., 2006). The working model (referred to as model M1) must also include terms which partition residual variance for those traits where the experimental unit is not the observational unit (Bailey, 2008). These traits include internal infection, for FT17, FT18, and FT19; internal infection and mortality for SH16, and SH17; and UCI and plant height for FT18 and FT19. All models also include a term representing polygenic effects, and the integrity of the experimental designs are preserved by including an additional term as a fixed effect, representing the effects of those accessions which were not genotyped but had phenotypic data (Tolhurst et al., 2019). All trials and traits were analyzed individually except the two runs of the shade-house experiments SH16 and SH17 which were analyzed jointly considering years as “environments”.

The models were fitted either as a linear mixed model (LMM) using residual maximum likelihood (REML) (Patterson and Thompson, 1971) for the traits internal infection, UCI, days to flower, plant height and maturity which were considered to be approximately Gaussian on a suitable transformed scale, or as a generalized linear mixed model (GLMM) using penalized quasi likelihood (Breslow and Clayton, 1993) for the traits: plant survival and mortality. All analyses were conducted using *ASReml-R* (Butler et al., 2018), which provides REML estimates of variance parameters, empirical best linear unbiased predictions (EBLUPs) of random effects and empirical best linear unbiased estimates (EBLUEs) of fixed effects.

Heritability (reliability) for each experiment and trait was computed using a generalized definition for heritability developed using the methods of Cullis et al. (2006):

$$h^{2}=1-\;PEV/\left(2\theta_{gt}^{2}\right)$$

where *PEV* is the average pair-wise prediction error variance of genotype effects and $\theta_{gt}^{2}$ is the genetic variance of trial *t*. Pair-wise marker additive genetic correlations (*r_a_*) between traits were computed using the EBLUPs of the marker additive genetic effects of those accessions evaluated for each trait.

### GWAS Approach

Using the working model, M1, genome scans for each linkage group with a positive REML estimate of the marker variance was performed. During this scan, each marker was fitted as a fixed effect, the other markers on the same linkage group were dropped from the model, the marker variances for the other linkage groups were held fixed at the values from the base working model while all other variance parameters were re-estimated. Potential QTLs were chosen from this scan, along with their positions and LOD scores, i.e., –*log*_10_(*P* – *value*). The potential set of markers was then thinned using a modification of the approach of Benjamini and Hochberg (1995). Given the strong dependence of the tests within a linkage group, the squared LD (r^2^) was computed for each pair of markers and any markers with an estimated LD of greater than 0.7 and within 100 kb, were deemed to be within the same linkage block. Markers which were in the same linkage block and also chosen as potential QTLs were thinned again, to leave only one potential QTL within any given linkage block. The linkage block marker was chosen as the marker with the lowest *P*-value and the least number of missing values, for that linkage block. The remaining set of markers obtained from this were then included as the baseline multi-QTL model which included all of the same terms as the working baseline model as well as the set of QTLs fitted as fixed effects. Markers were dropped from this baseline multi-QTL model using a Bonferroni based backward elimination approach until all remaining markers were significant at a significance level of 0.05. All remaining markers in the fixed component of the final multi-QTL model are reported here as putative QTLs. Of which the markers with *P*-value ≤ 0.001, i.e., LOD score ≥ 3 are considered as “significant,” and the markers with 0.001 < *P* value < 0.05 are considered as “suggestive” (Rexroad et al., 2012).

### Putative Candidate Genes for Resistance

Marker sequences that revealed significant associations with quantitative resistance to blackleg in this GWAS study and previous studies (Jestin et al., 2011; Fopa Fomeju et al., 2014; Fopa Fomeju et al., 2015; Larkan et al., 2016a; Raman et al., 2016; Kumar et al., 2018) were aligned by searching their physical positions with the reference Darmor-*bzh* gene assembly version 4.1 (Chalhoub et al., 2014). Biological functions of those genes in relation to *R* and QR loci were described.

### Comparative Mapping of SNP Associations in Relation to AK of Brassicaceae and Disease Resistance *R* Genes

Marker sequences of significant SNP associations for resistance were aligned with the sequence of *A. thaliana* to identify the location of QR loci and its relationship with AK of Brassicaceae, as described previously (Zou et al., 2016). *Brassica napus* *R* genes (Chalhoub et al., 2014; Alamery et al., 2018) that map in the vicinity of genomic regions associated with resistance to blackleg were also searched for their synteny with AK blocks.

## Results

### Genetic Variation for Resistance to Blackleg

**Figure 1:**
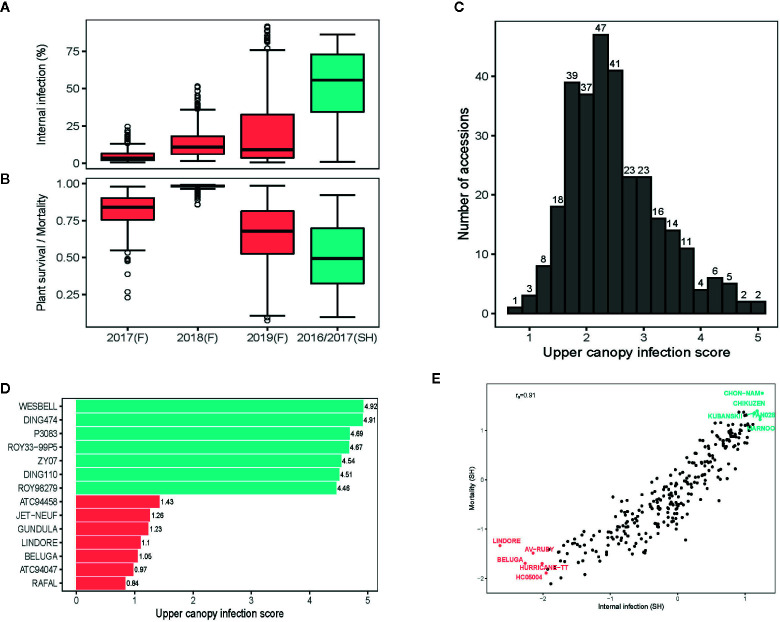
Variation for resistance to blackleg disease, caused by the fungus, *Leptosphaeria maculans* in canola. Box plots showing variation for resistance to blackleg disease, measured as per cent internal infection **(A)** and as plant survival/mortality **(B)** assessed across five environments. In total, disease severity scores from 421 diverse accessions are evaluated across five environments (for details of accessions evaluated, see **Supplementary Table 1**). **(C)** The frequency distribution of upper canopy infection scores among diverse accessions. **(D)** Accessions showing extremes in resistance and susceptibility to upper canopy blackleg infection, evaluated across two field environments (2018, 2019) at Wagga Wagga, NSW. Variation for blackleg severity was assessed under field conditions (in blackleg nurseries) and under shade-house conditions (with ascospore shower method). **(E)** Scatter plots showing relationship between plant mortality and internal infection among diverse accessions, assessed for resistance using ascospore shower test. Stubble collected from commercial crops of Australian canola cultivars: ATR-Gem (group A; Cudal, NSW), CB-Telfer (group B, Arthurton, South Australia), ATR-Stingray (group C, Minyip, Victoria), Hyola450TT (group ABD, Streatham, Victoria), Hyola650TT (group ABD, Grenfell, NSW), T28156 (group F, unreleased breeding line, Parkes, NSW) and ATR-Marlin (group S, Parkes, NSW) grown in 2015, was used for “ascospore shower.” Empirical best linear unbiased predictions (EBLUPs) of genetic effects are plotted.

Despite of the extreme high day-temperatures and drought conditions across field environments (**Supplementary Figure 2**), we were able to raise blackleg nurseries, suitable for evaluation of germplasm for resistance. Frequent overhead watering, with the lateral move irrigator ensured that condition for blackleg infection were congenial throughout the growing season. Across environments, blackleg infection occurred, right from the cotyledon stage (after 4–6 weeks of seedling emergence) to silique maturation (**Supplementary Figure 3A**). The maximum disease expression for UCI was observed at the silique maturation stage, when the disease severity for UCI was assessed. Frequency distributions exhibited that severity of blackleg scores, measured as internal infection and plant mortality, were variable across environments; blackleg severity was higher under shade-house (ascospore shower test) compared to disease nurseries under field conditions (**Figures 1A, B**).

Joint analysis of the two shade-house experiments, where accessions were evaluated with the “ascospore shower” method, revealed that the majority of them were susceptible to pathotypes, present on the infested stubble derived from the Australian canola cultivars: ATR-Gem, CB-Telfer, ATR-Stingray, Hyola450TT, Hyola650TT, T28156 and ATR-Marlin (**Supplementary Table 3**). We identified 21 accessions of canola, and one accession of Ethiopian mustard (*B. carinata*, *2n =4x =34*, subgenome B_c_B_c_C_c_C_c,_ ATC93184) that had ≤10% internal infection in the shade-house experiments (**Supplementary Table 3**). Resistant *B. napus* accessions were of Australian (43C80CL, 46C04, AGA99, ATR-Beacon, ATR-Mako, ATR-Signal, AV-Ruby, BononzaTT, HurricaneTT, ScaddanTT, Surpass501TT, ThunderTT, WarriorCL, RQ01-02), European (Global, Columbus, Beluga, Lindore) and Asian (Gan-You and Tosharsu) origins. The level of resistance in 16 accessions was similar to contemporary and modern commercial cultivars: GT42 (*Rlm1,4,6)*, Hyola575 (*LepR1, Rlm1*) and Hyola970 (*Rlm7*) (**Supplementary Table 3**). Several cultivars, carrying the race-specific resistance genes, such as Stingray (*Rlm3*,9), Ripper (*Rlm2,4,9*) and CB-Telfer (*Rlm4*) were susceptible under shade-house and field conditions, suggesting that major genes were ineffective in conferring resistance, while some of the resistant accessions such as Scaddan (*Rlm1,4*), Bonanza (*Rlm2,9*) and ThunderTT (*Rlm4,9*) had a very low scores for internal infection when challenged with ascospores released from cultivars carrying *Rlm1, Rlm2, Rlm4 and Rlm9* genes (either single or in stack), hinting that those accessions may have loci for quantitative disease resistance or a combination of both novel *R* and QR loci, this requires further verification. Diverse panel of canola accession showed a continuous distribution of disease severity scores for UCI, suggesting that multiple genes control resistance to UCI (**Figure 1C**). Among different accessions, Beluga, Gundula, Jet Neuf - a donor source of quantitative resistance in Darmor-*bzh* and other winter cultivars (Pilet et al., 2001), Lindore, Rafal, and two accessions of *B. carinata* (ATC94047, ATC94458) were highly resistant to UCI, while P3083, Ding474 and Wesbell were rated as highly susceptible (**Figure 1D**).

Our analysis showed that the significant source of genetic variation in blackleg resistance was from the additive component, i.e., genetic markers; VAF_a_, which ranged from 33.8% for plant survival (FT17) to 100% for internal infection (SH17, FT17) and mortality (SH17) (**Table 1**). The values for the reliabilities (broad sense genomic heritability) for each trait measured were generally high (**Table 2**) and found to be trait and environment dependent. For example, plant survival had low reliability (42%) in 2018, but higher values were obtained in 2017 (87%) and in 2019 (99%) environments. Flowering time had higher values for reliability across all environments (97% to 98%) compared to other traits. High reliability values suggest that the variation in different traits measured is heritable and therefore is suitable for genetic analysis as well as for exploiting in the canola breeding programs for enhancing genetic gains.

**Table 1:** Summary of the residual maximum likelihood (REML) estimates for additive and nonadditive (residual) genetic variance from the models before (Base marker model: M1) and after identifying putative quantitative trait loci (QTL) (Final multi QTL model: M2) for each of the trait and trial.

Trait	Experiment	Additive (M1)	Additive (M2)	Nonadditive (M1)	Nonadditive (M2)	VAF_a_ (%)	VAF_m_ (%)
Internal infection	SH16	1.46	0.91	0.27	0.30	84.4	28.3
Internal infection	SH17	5.21	2.81	0.00	0.00	100.0	43.6
Internal infection	SH16/17	2.68	1.66	0.17	0.10	94.0	37.0
Internal infection	FT17	0.86	0.64	0.00	0.00	100.0	22.6
Internal infection	FT18	1.35	0.67	0.04	0.00	97.4	51.8
Internal infection	FT19	3.58	2.48	0.750	0.75	82.7	28.5
UCI	FT18	0.27	0.06	0.17	0.19	61.2	47.7
UCI	FT19	0.24	0.06	0.05	0.07	81.5	59.0
Height	FT18	129.75	46.42	51.53	55.69	71.6	43.2
Height	FT19	117.81	63.68	56.17	53.41	67.7	31.2
DTF	FT17	36.50	20.90	5.09	5.12	87.8	34.4
DTF	FT18	158.48	68.18	13.41	13.96	91.1	48.9
DTF	FT19	48.56	23.67	7.12	7.06	87.5	39.8
Plant survival	FT17	0.31	0.27	0.61	0.58	33.8	7.8
Plant survival	FT18	0.67	0.42	0.23	0.12	74.1	38.7
Plant survival	FT19	0.57	0.42	0.57	0.44	49.7	27.2
Mortality	SH16	0.68	0.32	0.21	0.16	76.0	46.9
Mortality	SH17	2.79	1.92	0.00	0.00	100.0	27.8
Mortality	SH16/17	1.04	0.55	0.19	0.21	84.6	35.6

VAF_a_ is the percentage of total genetic variance accounted by the markers. VAF_m_ shows the percentage of genetic variance accounted by the identified putative QTLs. Resistance to blackleg was assessed as internal infection (stem canker, %) and plant survival/mortality (0–1) across five environments in 2016, 2017, 2018, and 2019. SH, shade-house; FT, Field; DTF, Days to flower; UCI, Upper canopy infection (1-5 scale); SH16/17, joint analysis of SH16 and SH17.

**Table 2:** Summary of heritability, mean, minimum and maximum values of the predicted means for each trait and trial in the diverse panel of canola accessions.

Phenotypic Environment	Year	Trait	Trial	Heritability (%)	Mean	Minimum	Maximum
Shade-house	2016	Internal infection	SH16	80	83.79	11.67	98.88
	2017	Internal infection	SH17	84	74.26	3.81	98.84
	2016	Mortality	SH16	72	0.46	0.13	0.85
	2017	Mortality	SH17	77	0.34	0.06	0.90
	2016/2017	Internal infection	SH16/17	81	51.89	0.82	86.35
	2016/2017	Mortality	SH16/17	75	0.50	0.10	0.92
Field	2017	Internal infection	FT17	66	4.75	0.55	24.47
	2018	Internal infection	FT18	67	22.45	0.60	91.84
	2019	Internal infection	FT19	81	22.45	0.60	91.84
	2018	UCI	FT18	74	1.99	0.18	4.41
	2019	UCI	FT19	76	1.76	0.34	4.45
	2018	Height	FT18	89	128.98	91.86	160.44
	2019	Height	FT19	90	90.35	28.46	123.36
	2017	DTF	FT17	98	120.39	109.20	139.57
	2018	DTF	FT18	98	122.17	102.95	155.60
	2019	DTF	FT19	97	115.42	100.81	139.35
	2017	Plant survival	FT17	87	0.81	0.23	0.98
	2018	Plant survival	FT18	42	0.98	0.86	0.99
	2019	Plant survival	FT19	99	0.66	0.08	0.98

Resistance to blackleg was assessed as internal infection (%) and plant survival/mortality (proportion) across five environments in 2016, 2017, 2018, and 2019. SH, shade-house; FT, Field; DTF, Days to flower; UCI, Upper canopy infection (1–5 scale). Data on SH16/17 is based on the joint analysis of SH16 and SH17 trials.

### Genetic Correlation Between Blackleg Traits

**Figure 2:**
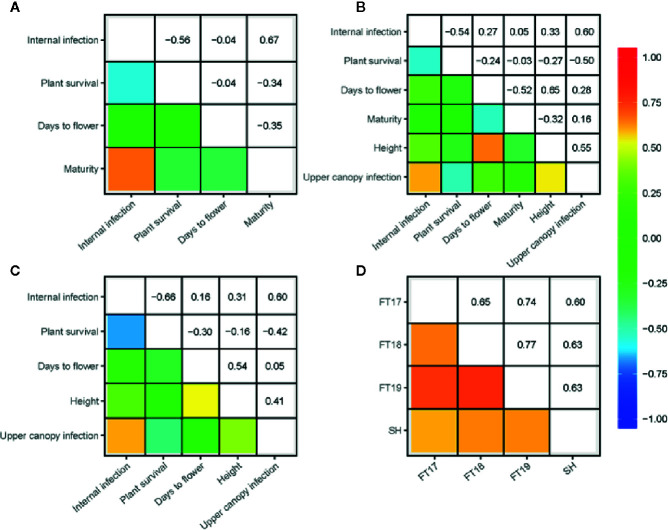
Genetic (additive) correlations for resistance to blackleg evaluated in disease nurseries across three environments in 2017 **(A)**, 2018 **(B)**, and 2019 **(C)** at Wagga Wagga, NSW, Australia. Durability of resistance (assessed as internal infection) across three field experiments and shade-house (joint analysis of 2016 and 2017 experiments) also shown **(D)** Blackleg severity was evaluated on the basis of plant survival (proportion), internal infection (crown canker, %) and upper canopy infection (UCI, 1–5 scale) among diverse accessions (for details, see **Supplementary Table 1**). Plant development traits: plant height (cm) was measured for five plants per plot; maturity was scored for each plot; flowering time (days to first flower, GS 65) was determined, when 50% of plants have first open flower from the day of sowing as days to flower. Pair-wise correlations (r_a_) between traits were computed using the EBLUPs of the marker additive genetic effects.

We investigated the pair-wise additive genetic correlation between different measures of blackleg scores among accessions. There was a high positive correlation (*r_a_* = 0.91) between internal infection and mortality in ascospore shower test (**Figure 1E**). Likewise, internal infection scores showed negative correlation with plant survival in 2017 (*r_a_* = −0.56), 2018 (*r_a_* = −0.54) and in 2019 field environments (*r_a_* = −0.66) (**Figures 2A–C**). Resistance to UCI, evaluated across two years also showed a negative correlation with plant survival across both environments (2018*; r_a_* = −0.50, 2019; *r_a_* = −0.42). To determine the effectiveness of quantitative resistance across environments (field and shade-house), we further investigated the relationship between internal infection (stem canker) scores among accessions. We observed a high additive genetic correlation (*r_a_* = 0.60 to 0.77), suggesting that diverse accessions have effective resistance to blackleg (**Figure 2D**).

### Genetic Control of Blackleg Resistance

**Figure 3:**
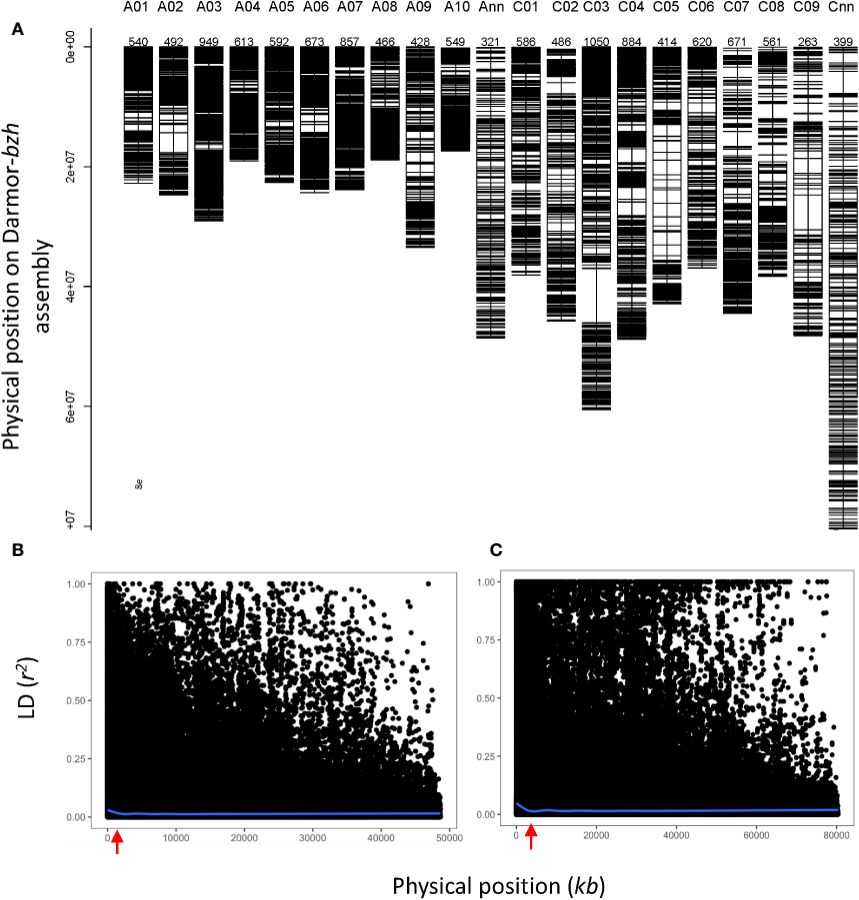
Distribution of SNP markers **(A)** and LD (*r* ^2^) among diverse accessions of canola across 19 linkage groups of *B. napus* (A01-C09) and unanchored An_random (Ann) and Cn_random (Cnn) linkage groups. LD decay across the A_n_ subgenome **(B)** and C_n_ subgenome **(C)**. LD decay occurs (*r* ^2 ^= 0.2) approximately 0.61 Mb and 1 Mb in A_n_ and C_n_ subgenomes, respectively. The *r* ^2^ is estimated for each pair of SNPs used for GWAS.

To gain an understanding of genetic loci controlling variation in QR against *L.* *maculans*, we performed GWAS analysis. This was accomplished by using 12,414 genome-wide distributed SNPs across all 19 linkage groups (and Ann_random and Cnn_random linkage groups) of *B. napus* (**Figure 3A**). Marker density on the A_n_ subgenome was higher (n = 6,480) compared to the C_n_ subgenome (n = 5,939) and covered 0.77 Gb of the reference Darmor-*bzh* genome (**Supplementary Table 4**). The LD was estimated at the whole genome level, using the full set of SNP markers, for each chromosome and both A_n_ and C_n_ subgenomes (**Figures 3B, C**, **Supplementary Figure 4**). The LD decay (*r* ^2^ = 0.2) of the A_n_ and C_n_ subgenomes varied from 0.6 Mb to 2 Mb, respectively. After thinning markers controlling for false discovery rate, and accounting LD among diverse accessions, GWAS revealed 26 highly significant (LOD score of ≥3, *P* ≤ 0.001) and 33 “suggestive” (LOD score of <3, 0.001 < *P* < 0.05) associations for resistance to *L. maculans*, scored as per cent internal canker infection, plant survival and UCI on 17 chromosomes of canola, with the exception of C07 and C08 (**Supplementary Table 5**). SNP markers explained 7.8 to 59.0% of genetic variance for resistance to blackleg (**Table 1**).

#### SNP Associations Detected for Resistance Using “Ascospore Shower” Screens

**Figure 4:**
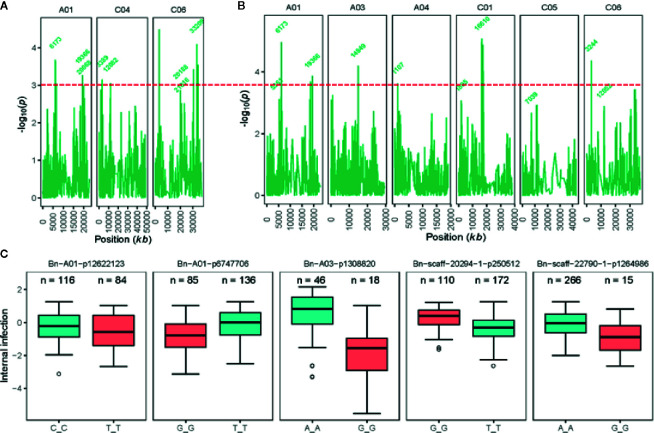
Genetic loci associated with resistance to blackleg, assessed using “ascospore shower test” in diverse accessions of canola. Manhattan plots showing LOD scores (-log_10_ *P*) for association between Illumina SNP and disease severity: plant mortality **(A)** and internal infection **(B)** in 285 (genotyped) of 327 canola accessions (for details, see **Supplementary Table 1**). LOD scores presented in the Manhattan plots are from Base marker model M1. The red dash line indicates the threshold value for highly significant SNPs at LOD ≥ 3. Positions (kb) of the highly significant (LOD ≥ 3, P ≤ 0.001) and “suggestive” (LOD < 3, 0.001 < P < 0.05) markers from the final multi-QTL model (M2) are labeled. The physical positions of SNPs are based on the map position on the Darmor-*bzh* gene assembly (Clarke et al., 2016, **Supplementary Table 5**). **(C)** Box plot showing the distribution of the EBLUPs for internal infection partitioned into allele combinations for the SNP markers that are significantly associated with resistance (LOD ≥ 3) assessed under shade-house conditions with “ascospore shower” test in a diverse panel of GWAS accessions. Major (present in high proportion) and minor (present in low proportion) allele for each marker are shown in different colors. Number of alleles (n) for each significant SNPs associated with resistance are also shown. Distributions of SNP marker Bn-A03-p13088220 allele represent to SH17 experiment, where 68 canola accessions were evaluated for resistance to blackleg using the “ascospore shower test.”.

Joint analysis of the two shade-house experiments identified 18 (four significant and 14 suggestive) SNP associations for resistance against pathotypes present on stubble of groups A, B, C, D, E, F, and S commercial canola cultivars (**Table 3**; **Supplementary Table 5**). For plant mortality, eight SNP associations, comprising two significant and six “suggestive,” were identified on chromosomes A01, C04, and C06 (**Figure 4A**, **Supplementary Table 5**). The maximum genetic variance (9.1%) was explained by each of the two “suggestive” SNPs, Bn-A01-p6747706 (on A01) and Bn-scaff_16576_1-p256367 on chromosome C04. For internal infection, 10 SNP associations; two significant and eight “suggestive,” were identified on chromosomes A01, A03, A04, C01, C05, and C06 (**Figure 4B**). The maximum genetic variance (8.3%) was explained by the Bn-scaff_15838_1-p1403775 SNP on C01, followed by Bn-scaff_17088_3-p242403 on C06 (6.4%). As both mortality/plant survival and internal infection (stem canker) phenotypes were highly correlated (*r_a_* = 0.91, **Figure 1E**), we identified two genomic regions that were collocated on chromosome A01 (**Supplementary Table 5**). To determine the effect of population size on marker-trait association, we undertook GWAS analysis for each environment independently of ascospore shower screens, conducted across the two experiments (SH16 and SH17). In the SH16 experiment, four significant SNP associations were identified: two were identified for resistance to internal infection on chromosome A01 within genomic interval of 6.17 Mb to 10.87 Mb, while, four SNP associations were identified for plant survival on A01, A03, and C06 (**Table 3**, **Supplementary Table 5**). In SH17 experiment, two significant SNP associations were identified: the first for internal infection on A03 (0.94 Mb), and second for plant survival on A06 (1.58 Mb) and none of them were detected in SH16 (**Supplementary Table 5**). This discrepancy was likely due to the effective population size affecting both population structure as well as local LD among accessions. Overlaying results from single experiments (SH16 and SH17) with from joint analysis (SH16/SH17), we found that two regions for resistance on A01 (5.3 Mb; 6.17 Mb); one region on A03 (14.8 Mb); and one region on C06 (33.2 to 33.5 Mb, within LD) were detected across individual and joint analyses (**Supplementary Table 5**).

**Table 3:** Genome-wide association analysis showing significant statistical association between Illumina SNP markers and resistance to *L. maculans*.

Trait	SNP marker	Experiment	Linkage group	Position (bp)	*P*-value	LOD	*R* ^2^ (%)
**Mortality**	**Bn-A01-p6747706**	**SH16**	**A01**	**6173561**	**0.001**	**3.11**	**9.6**
**Internal infection**	**Bn-A01-p6747706**	**SH16**	**A01**	**6173561**	**0**	**3.33**	**6.5**
Internal infection	Bn-A01-p12622123	SH16	A01	10872512	0.001	3.27	5.1
Plant survival	Bn-A02-p27743910	FT18	A02	24724645	0.001	3.02	9.7
Internal infection	Bn-A03-p1308820	SH17	A03	945040	0.001	3.01	16.5
Internal infection	Bn-A03-p4352837	FT19	A03	3885180	0	3.59	6.9
Upper canopy infection	Bn-A03-p8475614	FT19	A03	7780069	0	5.05	19.2
Mortality	Bn-A03-p15815965	SH16	A03	14892225	0	4.26	10.1
**Upper canopy infection**	**Bn-A04-p2448580**	**FT19**	**A04**	**2133626**	**0**	**5.97**	**23.4**
**Upper canopy infection**	**Bn-A04-p2713564**	**FT18**	**A04**	**2419904**	**0**	**3.51**	**10.2**
Upper canopy infection	Bn-A04-p4099958	FT19	A04	4169810	0	4	11.7
Upper canopy infection	Bn-A05-p22141046	FT19	A05	20252157	0	6.73	25.1
Mortality	Bn-A06-p18000461	SH17	A06	1585510	0	3.8	8
Plant survival	Bn-scaff_15705_1-p84236	FT19	A06	19564715	0	4.53	2.9
Internal infection	Bn-A07-p11185122	FT18	A07	12477068	0	3.46	13.5
Upper canopy infection	Bn-A02-p771951	FT18	A07	13400889	0.001	3.01	7
Internal infection	Bn-A07-p16036626	FT19	A07	17951152	0	4.57	8
Upper canopy infection	Bn-A07-p18226326	FT18	A07	20118101	0.001	2.96	11.6
Internal infection	Bn-A09-p10092753	FT17	A09	9086363	0	4.17	13.9
Internal infection	Bn-A09-p20157367	FT18	A09	17156001	0	3.73	10.8
Plant survival	Bn-A10-p2048923	FT19	A10	1671039	0	3.4	6.3
Internal infection	Bn-A10-p15021776	FT18	A10	14967128	0	4.7	13.3
Plant survival	Bn-scaff_15838_1-p790905	FT18	C01	1224850	0.001	3	11.9
Internal infection	Bn-scaff_22790_1-p1264986	SH16/17	C01	16610817	0	4.99	3.1
Plant survival	Bn-scaff_15712_6-p292471	FT17	C02	37086215	0	5.01	7.7
Internal infection	Bn-scaff_18917_1-p533499	FT17	C03	29919378	0	3.51	10.9
Internal infection	Bn-A05-p17894045	FT18	C05	33067591	0	4.11	13.5
Internal infection	Bn-scaff_20294_1-p250512	SH16/17	C06	3244625	0	3.39	3.4
Mortality	Bn-scaff_15746_1-p145442	SH16/17	C06	21016294	0	3.53	7.5
Mortality	Bn-scaff_23957_1-p151052	SH16	C06	30618173	0.001	3.26	3.5
**Mortality**	**Bn-scaff_25466_1-p33838**	**SH16/17**	**C06**	**33209209**	**0**	**3.51**	**6.3**
**Mortality**	**Bn-scaff_20294_1-p395143**	**SH16**	**C06**	**33566786**	**0**	**3.86**	**22**
Internal infection	Bn-scaff_17554_1-p19888	FT19	C09	39720977	0	4.3	8.1
Upper canopy infection	Bn-scaff_15576_1-p115266	FT19	C09	41180043	0.001	3.27	14
Upper canopy infection	Bn-scaff_16361_1-p1072715	FT18	Cnn_random	62936531	0	4.33	12.8

Resistance was assessed as plant mortality (proportion) and internal infection (%) using “ascospore shower” test under shade-house conditions across 2016 (SH16) and 2017 (SH17) whereas it was assessed as plant survival (proportion), internal stem infection (%) and upper canopy infection (1–5 scale) across three field environments (2017: FT17, 2018: FT18, and 2019: FT19). Only markers that revealed the LOD score ≥3 are shown herein. Marker associations with LOD scores of <3 are given in a **Supplementary Table 5**. The physical positions of SNPs are based on the map position on the Darmor-bzh gene assembly (Clarke et al., 2016). Associations in bold represent to those which appeared across experiments and in LD. The LD was estimated in the diverse panel of canola accessions. R^2^ and SH16/17 refer to the percentage of genetic variance explained by the SNP marker and joint analysis of SH16 and SH17 experiments, respectively.

To identify SNP markers suitable for tracking QR genes in *B. napus* germplasm, we compared relationship between marker alleles that showed significant associations to *L. maculans* with “ascospore shower” and variation in resistance to blackleg, assessed by the severity of internal infection. Our analysis showed that single SNP association could not sufficiently account for resistance. However, using multiple alleles for different QTL regions, resistance could be tracked in a diverse panel of canola accessions (**Figure 4C**). For example, SNP alleles present in a greater proportion (TT, Bn-scaff-20294-1-p250512) and in smaller proportion on chromosomes A01 (GG, Bn-A01-p6747706), A03 (GG, Bn-A03-p1308820), and C01 (GG, Bn-scaff-22790-1-p1264986), contributed in increasing resistance to internal infection under shade-house conditions.

#### SNP Associations for Plant Survival in Field

**Figure 5:**
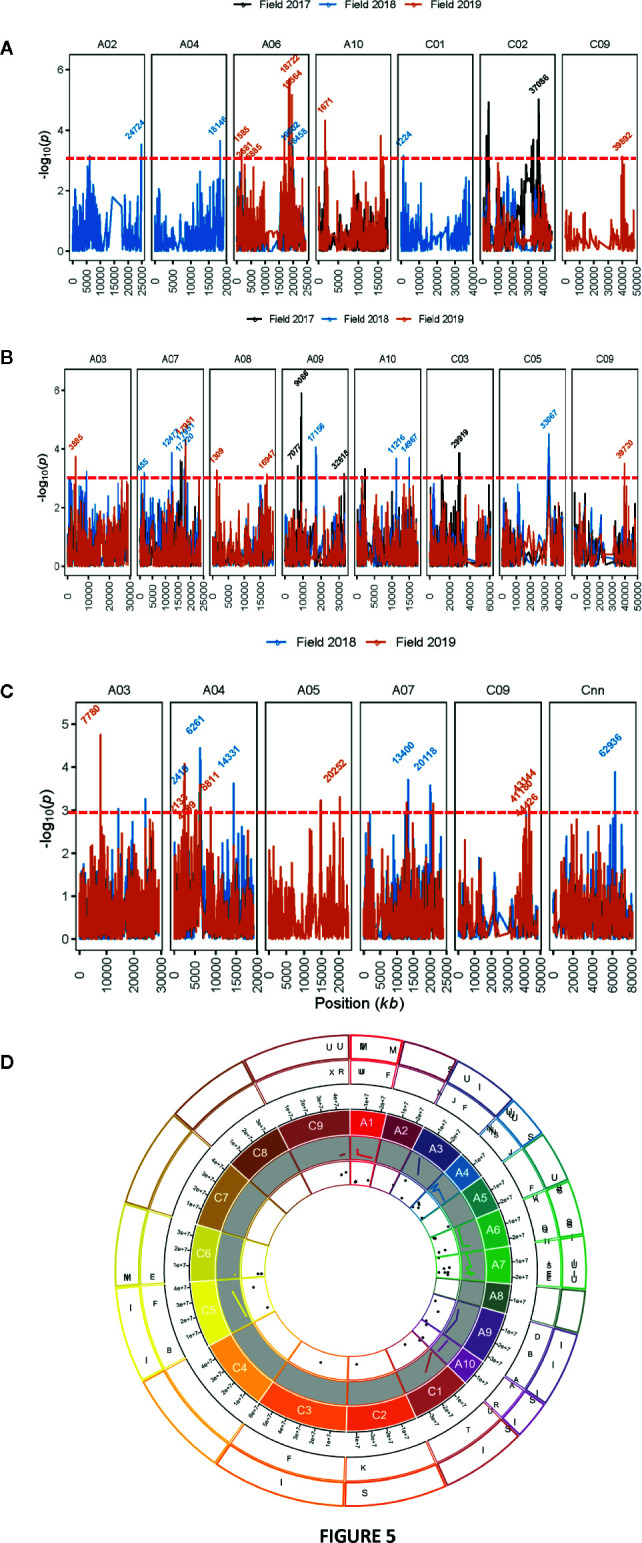
Manhattan plots showing genomic regions associated with resistance to blackleg in diverse accessions. Resistance was assessed by plant survival **(A)**, internal infection **(B)** in blackleg nursery across three field environments in three consecutive years (2017 to 2019) at Wagga Wagga, while upper canopy infection **(C)** was assessed across two years (2018 and 2019). LOD scores (-log_10_ *P*) for association between Illumina SNP and disease severity in diverse canola accessions are shown on y-axis. LOD scores presented in the Manhattan plots are from Base marker model M1. The red dash line indicates the threshold value for significant SNPs at LOD ≥ 3. Positions (kb) of the highly significant (LOD ≥ 3, P ≤ 0.001) and “suggestive” (LOD < 3, 0.001 < P < 0.05) markers from the final multi-QTL model (M2) are labeled. The physical positions of SNPs (x-axis) are based on the map position on the Darmor-*bzh* genome assembly (Clarke et al., 2016, for detail, see **Supplementary Table 5**). **(D)** Circos plot with 19 chromosomes showing localization of quantitative trait loci (QTL) associated with resistance to blackleg and phenological components in a diverse panel of canola accessions on the 19 chromosomes of canola. Physical positions of SNP markers were inferred on the positions of the Darmor-*bzh* reference genome assembly. Scatter plots and lines represent LOD scores and R^2^ values of SNP associations for different traits evaluated in a GWAS panel. Lines represent to R^2^ values (inner most shell). Outer most shell represents to different traits (I: internal infection; S: plant survival, M: plant mortality, Mat: plant maturity; H: plant height; U: upper canopy infection; F: flowering time), evaluated in a GWAS panel. The second outer most shell illustrates the ancestral blocks. Localization of SNP markers associated with resistance to blackleg is only shown on the ancestral blocks of Brassicaceae, as defined by Schranz et al., 2006 by aligning *A. thaliana* genes to the Darmor reference genome.

GWAS identified 13 SNPs (five significant and eight “suggestive”) that were associated with resistance to *L. maculans,* based on the plant survival scores under field conditions across three environments (**Figure 5A**, **Supplementary Table 5**). Of the five significant associations (LOD ≥3) identified on A02, A06, A10, C01, and C02 chromosomes, the SNP Bn-scaff_15838_1-p790905 on C01 explained the maximum genetic variance (11.9%) for plant survival, followed by Bn-A02-p27743910 (9.7%) on A02 (**Table 3**). In total, GWAS associations explained 7.8% (2017) to 38.7% (2018) of the genetic variance (**Table 1**). Two genomic regions, the first delimited with two SNPs, Bn-A06-p17332795 and Bn-A06-p17042021, and the second region by the Bn-scaff_15705_1-p84236 and Bn-A06-p18432812 markers on A06, were detected within 0.26 Mb (in LD) across 2018 and 2019 environments (**Supplementary Table 5**).

#### SNP Associations for Resistance to Internal Infection in Field

Seventeen SNP associations (nine significant and eight “suggestive”) were identified for resistance to internal infection across three field environments (2017-2019) on chromosomes A03, A07, A08, A09, A10, C03, C05, and C09 (**Figure 5B**, **Supplementary Table 5**). SNP marker, Bn-A09-p10092753 on chromosome A09 accounted for 13.9% of genetic variation for internal infection (**Table 3**). Of the SNP associations, 82% of them (14/17) were mapped on the A_n_ subgenome, suggesting that the A genome is the richer reservoir of blackleg resistance genes compared to the C_n_ subgenome. This is consistent with earlier findings that majority of *R* genes (*Rlm1*, *Rlm2*, *Rlm3*, *Rlm4*, *Rlm7*, *Rlm9*, *Rlm12*, *LepR*1-4) identified so far, are mapped on the A_n_ subgenome, while only a few, *Rlm13* is mapped on the C_n_ subgenome (Raman et al, unpublished). Among SNP associations, only one genomic region, delimited with Bn-A07-p15812852 to Bn-A07-p16036626 (17.7 to 17.9 Mb sequence of Darmor-*bzh*) on chromosome A07 was detected repeatedly in LD across two field environments (FT18 and FT19; **Supplementary Table 5**).

#### SNP Associations for Resistance to UCI

Herein, we identified 14 associations (eight significant and six “suggestive”) for resistance against UCI on A03, A04, A05, A07, and C09 and Cnn_random contig of Darmor-*bzh* assembly (**Table 3**, **Figure 5C**). The number of SNP associations varied from 6 to 8, depending upon the phenotypic environment. Of the SNP associations, Bn-A05-p22141046 on A05 accounted for the maximum genetic variance (*R* ^2^ = 25.1%), followed by Bn-A04-p2448580 (*R* ^2^ = 23.4%) on A04. More than 42% of the genetic associations (6/14) were detected on A04, suggesting that multiple loci for resistance are localized on this chromosome. Across environments, one genomic region of the Darmor-*bzh* sequence (2.13 to 2.42 Mb); demarked with markers: Bn-A04-p2448580/Bn-A04-p2713564 was repeatedly detected in LD on A04 and accounted for up to 23.4% of the genetic variance (**Table 3**, **Supplementary Table 5**). Identification of multiple genomic regions associated with resistance, having small additive allelic effects, suggests that UCI is a quantitative trait and is controlled by multiple loci in canola.

### Hot-Spot Genetic Regions for Resistance to Blackleg

To identify hot-spot genomic regions for resistance to blackleg, we compared the physical positions of sequences of SNPs that reveal significant associations for plant survival, stem canker and UCI in diverse panel of canola accessions. The A_n_ subgenome had 39 SNP associations, while 20 associations were detected on the C_n_ subgenome (**Figure 5D**). Among the 19 linkage groups, A04, and A07 had 2.3 to 2.7 times more associations (QTL) for resistance to blackleg, compared to average genome-wide SNP (2.95/linkage group) associations (**Supplementary Figure 5**).

### Significantly Associated SNPs Overlap With Previously Identified QR and Known *R* Genes

**Figure 6:**
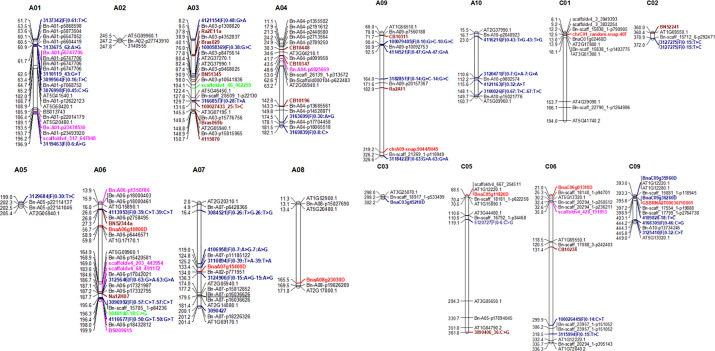
Comparison between genomic regions identified in a diverse panel of canola (this study) and previous studies. Marker loci highlighted in different colors represent to different studies. For clarity, we showed only the highly significant SNP associations for resistance (**Tables 3**and **4**) here. Candidate genes associated with *R* genes which map within 0.25 Mb are shown here. For details, see **Supplementary Table 6**.

To determine the uniqueness/redundancy of loci involved in QR resistance, we compared the physical positions of SNP associations identified in this study with the previous studies (Jestin et al., 2011; Fopa Fomeju et al., 2014; Larkan et al., 2016a; Rahman et al., 2016; Raman et al., 2016; Kumar et al., 2018; Raman et al., 2018; Raman et al., 2020). We identified several marker loci for QR to blackleg on chromosomes A01, A02, A03, A04, A05, A06, A07, A08, A09, A10, C01, C02, C05, and C06 that were mapped near the SNP associations identified in the present study (**Figure 6**). This suggests that QR loci identified here are relevant to international germplasm, comprising spring, semispring/winter and winter types. We also identified new QR loci on chromosomes A01 (10.8 Mb), A04 (1.1 Mb to 2.41 Mb), A06 (5.88 Mb), A08 (1.30 Mb, 16.94 Mb), A10 (1.67 Mb), C01 (1.80 Mb, 161.61 Mb), C03 (29.91 Mb), and C05 (7.03 Mb). These loci provide new targets for genetic improvement in canola breeding programs.

To identify plausible candidate genes in the QR genomic regions, we compared the physical locations of annotated *R* genes in the Darmor-*bzh* assembly (Chalhoub et al., 2014; Alamery et al., 2018). Forty-two *R* genes were located within 1 Mb of QR loci (**Supplementary Table 6**) of which 12 disease resistance *R* genes were located within 200 kb from the SNP associations (QTLs) on A01, A06, A07, A10, C01, C05; C06, and C09 (**Table 4**). These annotated *R* genes encode disease resistance proteins with the TIR-NSB-LRR and non-TIT-NBS-LRR (CNL), RLP and NB-ARC-LRR motifs, which are implicated in ETI (Jones and Dangl, 2006).

**Table 4:** Annotated disease resistance genes located in the vicinity of quantitative resistance to blackleg disease in canola grown across different environments.

Trait	SNP marker	Linkage group	Position	*P*-value	LOD	*R* ^2^ (%)	Candidate *R* gene			Distance from candidate gene	*R* gene motif
			(bp)				ID	Start	End	(bp)	
Mortality (SH16/17)	Bn-A01-p23905191	A01	20068468	0.007	2.19	4.40	BnaA01g28560D	19873137	19874480	195331	CC – NBS
Plant survival (FT18)	Bn-A02-p27743910	A02	24724645	0.001	3.02	9.66	BnaA02g36900D (A2_random)	24581776	24580933	142869	TIR-NBS-LRR
Plant survival (FT19)	Bn-A06-p18000403	A06	1585452	0.000	4.26	1.71	BnaA06g39650D (A6_ran) BnaA06g02940D	1655327 1789462	1658600 1792348	69817 204,010	CC-NBS-LRR
UCIS (FT18)	Bn-A02-p771951	A07	13400889	0.000	3.54	7.02	BnaA07g15400D	13339537	13343565	57,324	TIR-NBS-LRR
Internal infection (FT18)	Bn-A07-p16036626	A07	17951152	0.001	3.10	0.56	BnaA07g24250D BnaA07g24260D	18138909 18149353	18137486 18145885	193,122 187757	NBS - LRR TIR-NBS-LRR
Internal infection (FT18)	Bn-A10-p9802574	A10	11216126	0.001	3.22	4.78	BnaA10g13740D	11035464	11038549	177,577	NB-ARC domain-containing protein
Plant survival (FT18)	Bn-scaff_15838_1-p790905	C01	1224850	0.001	3.21	12.84	chrC01_random-snap.407 BnaC01g02460D	1246230 1302496	1244036 1303729	21380 77,646	TIR-NBS-LRR
Internal infection (SH16/17)	Bn-scaff_18181_1-p622258	C05	7039786	0.001	2.97	4.08	BnaC05g12130D	7046069	7053047	6,283	CC-NBS-LRR
Internal infection (SH16/17)	Bn-scaff_20294_1-p250512	C06	3244625	0.000	3.39	3.39	BnaC06g43800D (c06_random)	3240778	3,241,715	3847	TIR-NBS-LRR
Mortality (SH16)	Bn-scaff_20294_1-p395143	C06	33566786	0.002	2.69	16.03	BnaC06g34000D	33631584	33634387	67,601	TIR-NBS-LRR
Internal infection (FT19)	Bn-scaff_17554_1-p19888	C09	39720977	0.000	4.44	8.03	BnaC09g36260D	39561973	39561973	159004	NB-ARC-LRR, RLK
UCI (FT19)	Bn-scaff_15576_1-p115266	C09	41180043	0.001	3.28	14.03	BnaC09g38280D	41200106	41205204	20,063	Zinc finger

Only SNP associations that were detected within 200 kb are shown herein. Detailed information is presented in a **Supplementary Table 5**. Data on SH16 and SH17 experiments relates to ascospore shower test, while FT18, FT18, and FT19 experiments relate to field screening of GWAS panel. Data on SH16/17 is based on the joint analysis of SH16 and SH17 trials. R^2^ refers to the percentage of genetic variance explained by the SNP marker.

### Several QR Loci of Canola May Represent to Ancestral Genes of Brassicaceae

To determine and verify whether QR genomic regions are located in the homoeologous regions of *B. napus* genome, we searched the synteny between genomic regions that showed significant associations with QR, using SNP markers as proxies, and 22 AK blocks of Brassicaceae (Schranz et al., 2006). We delimited 37 SNP associations (identified on chromosomes A01, A02, A03, A04, A05, A06, A07, A009, A10, C001, C02, C3, C04, C05, C06, and C09) in the 17 AK blocks: A, B, C, D, E, F, H, I, J, K, N, O, Q, R, S, T, U, and X (**Figure 5D**). Six SNP associations, which were mapped on chromosomes A03, A04, A09, A10, C04 and C06 could not be located to the AK blocks (**Supplementary Table 7**). Of the 44 significant SNP associations for resistance, 25 (56.7%) were localized in the duplicated regions of the 9 AK blocks; the maximum SNP associations (n = 6) were located in each of the blocks: E, F, and U, while the minimum associations (n = 1) were identified in each of the B, D, H, I, K, and T blocks (**Supplementary Table 7**). In addition to the E, J, R, and U blocks identified for resistance to blackleg in European canola (Fopa Fomeju et al., 2015), we also mapped SNP association in the duplicated regions on A, B, C, D, F, H, I, K, N, Q, S, T and X AK (**Supplementary Table 7**). In this study, no SNP association for resistance could be assigned to the W block (Fopa Fomeju et al., 2014). In addition, one SNP association for resistance, assessed based on plant mortality on chromosome C04 could not be mapped onto the same ancestral karyotype J block, identified in a previous study (Fopa Fomeju et al., 2015).

We further investigated the locations of genomic regions for QR with subgenomic structure of AK blocks, determined on the level of fractionation (Parkin, 2011; Parkin et al., 2014). The maximum SNP associations were present in the least fractionated subgenome (LF = 18), followed by seven associations in the moderately fractionated subgenome (MF1) whereas, the minimum SNP associations (6) were in the most fractionated subgenome (MF2). Consistent with a previous study (Fopa Fomeju et al., 2015), one to six homoeologous regions for QR were identified in the LF, MF1, and MF2 subgenomes of duplicated regions of E, J, R, U, and W (**Supplementary Table 7**). For example, we identified SNP association for UCI to 39.93 Mb region on chromosome C09 (R block, LF subgenome). The same regions for QR was also identified at 39.93 Mb region on chromosome C09 in a *B. napus* GWAS panel of European origin (Fopa Fomeju et al., 2015). At least 18 disease resistance genes of *A. thaliana* (localized in AK blocks C, D, E, F, H, I, J, N, Q, R, S, U, and X) were located within 100 kb regions, suggesting that these *R* genes were retained in *B. napus* genome, despite of long term genome fractionation, evolution and domestication (Chalhoub et al., 2014; Parkin et al., 2014). This study and the previously reported ones revealed that majority of QR regions have originated as a result of homoeologous and paralogous exchanges occurred between *A. thaliana* and *B. napus* genomes.

### Relationship Between Resistance and Phenological Traits

Since resistance to blackleg seems to be conditioned with other plant developmental traits such as plant height and maturity (Pilet et al., 2001; Delourme et al., 2004; Huang et al., 2016; Raman et al., 2018), we scored these traits in the replicated trials conducted under field conditions (2017–2019). Pair-wise correlations of the additive genetic effects between different traits: blackleg resistance, flowering time, plant height and maturity are presented in **Figure 2**. Utilizing the data on the genetic variation in the developmental traits, we further (i) located loci associated with flowering time, plant height and maturity in the diversity panel and (ii) investigated their physical localization in relation to significant loci associated with resistance to blackleg. GWAS analysis identified 30 SNP associations for flowering time on chromosomes A01, A02, A03, A10, C03, and C07 (**Supplementary Table 5**, **Supplementary Figure 6**); at least one of them is in the vicinity of associations for QR to blackleg on A02 (24.6 to 24.7 Mb). This genomic region maps within 27.5 kb from the AGAMOUS-LIKE 69/MADS AFFECTING FLOWERING 4; MAF4, AT5G65070, 24.6 Mb on the Darmor assembly). Likewise, GWAS identified 13 SNP associations for plant height on chromosomes A02, A08, A10, C03, C06, and C07 (**Figure 5D**, **Supplementary Figure 6**). Of which, one marker association on A02 that was identified with flowering time (24.6 to 24.7 Mb), was also located in the same genomic region for plant survival in 2018 field environment (**Supplementary Table 5**). No QTL for plant height was detected on chromosome A06, where QR locus for blackleg was identified earlier in the Darmor-*bzh*/Yudal population (Huang et al., 2016; Kumar et al., 2018; Raman et al., 2018). Of the 14 SNP associations identified on A05, A08, A10, Ann_random, C02, C05 and C09 for plant maturity, possibly four genomic regions on chromosomes A08 (1.03 Mb to 1.30 Mb), A10 (1.61 to 1.67 Mb, and 14.6 to 14.9 Mb), and C09 (39.7 Mb) were detected near the SNP maker loci for resistance to blackleg (**Supplementary Table 5**). Moderate correlation between phenotypes and co-location of QTLs for resistance to blackleg and phenological traits (**Figures 2A–C** and **Figure 5D**) suggest that genes, underlying QTLs are either linked or may have pleiotropic allelic effects. It was difficult to measure causal relationship between plant development traits and resistance, as blackleg disease may have impacted their expression.

We looked if variation in flowering modulate blackleg infection. To understand this, we compared genetic variation in flowering time revealed in FT17 and FT18 experiments (this study) and previous experiments conducted at WWAI in the same environments (2017, 2018) on the same set of 300 diverse accessions (Raman et al., 2019). We observed a high correlation across environments (*r* = 0.98). The possibility of involvement of flowering time loci was also excluded by comparing the physical locations of SNP associations for flowering time, which were identified in the same set of GWAS panel evaluated under the similar field conditions, in the absence of blackleg disease; similar SNP-trait associations were detected, as found in experiments conducted in disease nurseries. This observation suggests variation in flowering time did not significantly affect blackleg disease in experimental conditions used in this study.

## Discussion

### Ascospore Shower and Field Screens Identify Additional Sources for Quantitative Resistance

In this study, we included a larger set of germplasm as compared to previous studies (Delourme et al., 2006; Kaur et al., 2009; Light et al., 2011; Raman et al., 2012b; Larkan et al., 2016a; Raman et al., 2016; Raman et al., 2018) and identified additional sources for resistance (**Figure 1C**, **Supplementary Table 3**). Optimal conditions for blackleg development are required for assessing variation in resistance. For example, under high disease pressure, it may be difficult to reveal variation in resistance to blackleg, as *L. maculans* is a hemibiotroph and can colonize on resistant accessions of canola and related species. We observed moderately high values for reliability, which are comparable to previous studies (Pilet et al., 1998; Raman et al., 2012b; Jestin et al., 2015; Larkan et al., 2016a; Raman et al., 2016; Raman et al., 2018; Raman et al., 2019b) (**Table 2**). Low reliability values (12%) observed in some environments could be due to empirical visual assessment of resistance and genotype x environment interactions, as observed in canola DH populations from Skipton/Ag-Spectrum and RP04/Ag-Outback and Darmor-*bzh*/Yudal (Raman et al., 2012b; Huang et al., 2016; Raman et al., 2020). Under moderate disease pressure, we could assess genetic variation in resistance to UCI to blackleg consistently across environments (reliability scores: 74 to 76%, **Table 2**). Hereby, we emphasize the genetic variation in resistance to UCI among the accessions of the diverse panel and report several sources of resistance and susceptibility (**Supplementary Table 3**). This information can be useful for (i) understanding the genetic basis of resistance to UCI in mapping populations as well as (ii) developing protocols for high-throughput phenotyping for comprehensive assessment of UCI. The resistant accessions can further be exploited in breeding programs for developing improved cultivars with overall resistance to blackleg in canola and its related species.

### Genome-Wide Distributed Loci Are Associated With Quantitative Resistance

We have delineated numerous loci for resistance to blackleg in a diverse panel of canola using a GWAS approach, although causative SNPs are not identified in this study. Majority of the Australian canola germplasm has either qualitative and/or quantitative “background” resistance. In fact, it is not common to find Australian cultivars that do not have either qualitative and/or quantitative resistance, as it has been the “backbone” trait of canola industry. Therefore, it is difficult to access Australian germplasm (with effective population size for GWAS analysis) that does not have *R* genes and utilize for the identification of QR genes comprehensively.

Most of the significant SNP associations identified in our screens do not correspond to the “known” *R* genes reported on chromosomes A01, A02, A06, A07, and A10 (Raman et al., 2013; Raman et al., 2016) and were not consistently detected across environments (**Table 3**). The results indicate that environment played a significant role in quantitative resistance expression. Similar observations were made in previous QTL and GWAS studies (Pilet et al., 2001; Jestin et al., 2011; Jestin et al., 2015; Huang et al., 2016; Kumar et al., 2018; Raman et al., 2018). Presence of SNP associations accounting low genetic variance (despite of moderate to high reliability, **Table 3**) on multiple genomic regions also indicate that QR is governed by quantitative genes (polygenic) rather than monogenic *R* genes, as the latter provide the complete resistance and account for larger proportion of genetic variance (Raman et al., 2012a; Raman et al., 2012b; Larkan et al., 2016b; Raman et al., 2018; Raman et al., 2020). We also observed that some SNPs accounted for variable LOD scores and genotypic variance (**Supplementary Table 5**). For example, Bn-A07-p16036626 on A07 (17.9 Mb) had “suggestive” LOD score of 1.82 in 2018, while the same SNP association had significant LOD score of 4.52 in 2019, suggesting that significance of trait-marker association relies on environment (phenotypic conditions including pathogen population) and “suggestive” associations should not be discounted in genetic improvement programs. More than 50% (32/59) of the SNP associations, detected on A01, A02, A03, A04, A05, A06, A07, A09, C02, C05, C06, and C09 chromosomes, were identified within 100 kb distance from the previously identified QTLs (**Supplementary Table 6**, **Figure 6**), at least in one of the mapping populations (Larkan et al., 2016a; Raman et al., 2016; Kumar et al., 2018; Raman et al., 2018; Raman et al., 2020). Other SNPs associations on A04, A10 and C01 were mapped 0.5 to 1 Mb from QTL identified in Aviso/Bristol, Canberra/Bristol, Darmor/Bristol, and Grizzly/Bristol populations (**Supplementary Table 6**). These results suggest the common QR loci for resistance to blackleg in the winter (Jestin et al., 2011) and diverse panel of *B. napus* germplasm utilized in this study. Unlike previous studies (Jestin et al., 2011; Larkan et al., 2016a; Kumar et al., 2018), significant QTL (LOD ≥3) on chromosome A08 could not be detected in our GWAS panel. However, there were “suggestive” associations (with LOD <3) on A08. This could be attributed to *L. maculans* population structure, environmental conditions and/or the origin of germplasm. Previous studies (Jestin et al., 2011; Fopa Fomeju et al., 2015; Kumar et al., 2018) utilized a smaller set of accessions of winter rapeseed for GWAS which were evaluated under European phenotypic environments, whereas herein we used a larger diverse set of germplasm comprising spring, semispring/winter and winter types under Australian growing environments.

Some of the SNP associations for QR to blackleg were present in the vicinity of known *R* genes (*Rlm3, Rlm4, Rlm7, and Rlm9)* on chromosomes A07 (17.7 to 17.9 Mb), *LepR3/Rlm2* on A10 (14.4 Mb), *LepR1* on A02, and *LepR4* on chromosome A06 (Yu et al., 2008; Yu et al., 2013; Larkan et al., 2016b; Raman et al., 2016; Raman et al., 2018). The 17.7 to 17.9 Mb genomic region of A07 represents to a cluster of *R* genes and maps approximately 2Mb apart the *Rlm9*, encoding Wall-Associated Kinase like protein (BnaA07g20220D, 15.91 Mb) that was recently cloned in canola. (Larkan et al., 2019). Previously, *Rlm9* was mapped to a 15.95 Mb region in a DH population from Darmor-*bzh*/Yudal population (Raman et al., 2018). The genetic region, delineated by the Bn-A07-p18226326 marker at the 20.1 Mb on the Darmor assembly for UCI could represent the *Rlm1* gene in the AK block, E (subgenome LF). It was interesting that the SNP associations on A07 (Bn-A07-p15812852/Bn-A07-p16036626); 17.7 to 17.9 Mb for resistance to internal infection (**Supplementary Table 5**) were not detected with ascospore shower test. This was due to the utilization of stubbles derived from cultivars having *Rlm1/Rlm3/Rlm4/Rlm7/Rlm9* and unknown QR genes, which likely have rendered *Rlm* genes on A07 ineffective, leading to no variation for genetic analyses to detect. Effectiveness of qualitative *R* genes under field conditions identified hereby is in contrast to earlier studies, where the effectiveness of *Rlm3*, *Rlm4*, and *Rlm9* was questioned under the Australian field conditions (Light et al., 2011; Raman et al., 2012a; Larkan et al., 2016a; Raman et al., 2016; Raman et al., 2018; Raman et al., 2020). This may have occurred due to fungal population structure and environmental conditions, which may have impacted the frequency and distribution of Avirulence genes in the *L. maculans* population in the disease nurseries. It is reiterated that field environments across 2017, 2018 and 2019 were less conducive for the development of high disease pressure (**Supplementary Figures 2A, B**), due to record excessive drought conditions and high temperatures (plus 40°C during day time).The strong effect of weather conditions including the temperature on the severity of blackleg (stem canker) is reported previously (Evans et al., 2008). Further work is required to established whether there is any interaction between QR and *R* genes (described above) and or QR identified under field conditions is due to the genetic effect of *R* genes (e.g. *Rlm12* and QTL region on A01; 5.35Mb to 6.33Mb on A02, *Rlm2/LepR3* and QTL region 14.6 Mb to 15.3Mb on A10; **Supplementary Table 6**).

Our findings suggest that the “ascospore shower” test using ascospores released from mixed stubble sources (from cultivars carrying *R* genes such as *Rlm1, Rlm4, Rlm6, LepR1*, and *LepR3*, used in this study) was found to be suitable for the identification of QR genes. In the present study, we identified several genomic regions for QR which were detected variably across environments. For example, we identified genomic regions for resistance on chromosome A01, including *Rlm12* under shade-house conditions, which were not detected under field conditions. These results suggest that complementary approaches should be explored for genetic analysis of QR to blackleg. Our findings and previous studies revealed that variation in quantitative resistance is due to quantitative genes, distributed genome-wide with smaller allelic effects, suggesting that variation in resistance has evolved in several independent genes in canola germplasm.

### GWAS Analysis Reveals Genomic Regions for Resistance to UCI

GWAS analysis has been extensively used to detect marker associations with trait of interest, especially which are in LD with QTL. Relying on this approach with genetic relationship estimates, we detected 16 associations between SNPs and UCI. Majority (56%) of the SNP associations were detected on chromosome A04. Detection of multiple SNPs in small genomic interval on A04 suggest that this region harbors gene(s) for resistance to UCI and is one of the candidates for fine mapping. In addition, SNP marker Bn-A03-p8475614, accounting for 19.2% of genetic variance was mapped in the proximity of QTL involved in resistance to blackleg (~180 kb) on chromosome A03 (7.59 Mb, Raman et al, 2016). In earlier studies, significant marker associations for resistance to both single spore isolates of *L. maculans* and stem canker were also detected on the homoeologous group 3 (A03/C03) chromosome (Jestin et al., 2015; Raman et al., 2016). Two genomic regions (13.4 Mb and 20.1 Mb) for resistance to UCI were identified on chromosome A07. The 13.4 Mb locus was mapped ~129.7 kb apart from the GWAS association for resistance at the cotyledon stage (Raman et al., 2016). One of the disease resistance genes, BnaA07g15400D (GSBRNA2T00098616001) was also located 57.3 kb proximal to this QR locus (**Supplementary Table 5**), implying that this genomic region is likely to be involved in resistance to blackleg. The 20.1 Mb genomic region for UCI may represent to the *Rlm1* gene. The physical position of *Rlm1* in the GWAS panel is consistent with our results on the mapping of *Rlm1* in a high-resolution mapping population of canola (Raman et al, unpublished). This current study also hints that the *R* gene mediated resistances to internal infection (mapped to the 17.7 Mb to 17.9 Mb of the Darmor-*bzh* sequence, *Rlm3,4/7/9*) and to UCI (mapped to the 20.11 Mb, *Rlm1*), are stable under high temperatures in field and is not prone to high temperatures. These results are in contrast with a previous study which reported that *Rlm6* gene is not stable under high temperatures (Huang et al., 2006a).

Consistent with our earlier and other GWAS studies (Rahman et al., 2016; Raman et al., 2016), we identified several significant associations for field resistance (disease nurseries in field and ascospore shower in shade-house) near genomic regions identified with single spore isolates. In addition, we detected genomic regions for QR near *R* genes, suggesting that mechanisms underlying host Pathogen-Associated Molecular Pattern triggered Immunity (PTI) and Effector-Triggered Immunity (ETI) are shared for qualitative and quantitative resistance (Thomma et al., 2011). Pathogens like *L. maculans* must overcome PTI to colonize on host and then to interact with ETI for defense response. It is not established here which *R*/*QR* loci are responsible for PTI or weaker ETI (Doehlemann and Hemetsberger, 2013). It is also possible that some of the genomic regions identified here could be associated with qualitative resistance due to (i) the genetic effects of *R* genes, (ii) ubiquitous presence of races of *L. maculans* in a canola crop and (iii) *Avr* genes that are in low frequency and not subjected to mutations, leading to virulence toward *R* genes.

### Possible Role of Plant Developmental Traits on Blackleg Resistance

There was a positive relationship between UCI, flowering time, maturity, and plant height in 2018, however no such consistent relationship was found in 2019. This could have been attributed to the water stress and high temperatures in 2019 (**Supplementary Figure 2**). Moderate temperatures and high humidity are known to favor blackleg disease (Ghanbarnia et al., 2009). One genomic region for plant survival on A02, demarked with markers: Bn-A02-p27327804 (24.6 Mb), Bn-A02-p27336483 (24.6 Mb) and Bn-A02-p27743910 (24.7 Mb) was mapped within 100 kb from the SNP associations for flowering time and plant height (**Supplementary Table 6**). These results suggest that phenology genes may have pleiotropic effects on the level of blackleg expression.

## Conclusions

In summary, we have delineated natural variation for resistance to blackleg in a diverse panel of canola. The specific accessions identified herein provide a useful resource for improving resistance to blackleg, and increasing genetic diversity in resistance genes, which can be exploited in the breeding programs. GWAS analysis identified 59 significant and “suggestive” loci for resistance to blackleg under shade-house and field conditions. These loci can only explain a small proportion of the genetic variance in resistance to blackleg; the sizable proportion of variance is accounted by G× E interaction, indicating that additional loci remain to be identified. Further research is required to assess the stability of QTL associations across canola growing regions in Australia and elsewhere. Given the adaptive nature of *L. maculans*, it is also important to demonstrate the uniqueness of genes conferring effective quantitative resistance to blackleg in *Brassica* species to expand the genetic base of canola - one of the most recent domesticated oil seed crops in recent history. As this and previous studies showed that QR is controlled by several genes with smaller allelic effects, introgression of a large number of loci is difficult *via* traditional marker-assisted selection (Fikere et al., 2018). However, favorable alleles for stable QR including for plant height and flowering time, which don’t confound the expression of QR can be enriched in the elite breeding lines using genomic selection approaches. Altogether with the results of previous studies, our results suggest that the gene network involved in QR is rather complex and evolved in a multifaceted manner to unleash genomic variation that contributed to natural variation in resistance to blackleg disease in canola.

## Data Availability Statement

The raw data supporting the conclusions of this article will be made available by the authors, without undue reservation.

## Author Contributions

HR conceived the research, planned experiments and wrote the paper. HR, BM, and RR conducted research and contributed to phenotyping. BC developed the statistical methods for GWAS analysis. RP performed statistical analysis and GWAS analysis in consultation with HR. SM and DB evaluated accessions for resistance using ascospore shower, in consultation with HR. YZ, SL, and HR performed comparative mapping of significantly associated SNP markers. All authors contributed to the article and approved the submitted version.

## Funding

This study was supported by the NSW Department of Primary Industries, the Grains Research and Development Corporation, and Agriculture Victoria Research (AVR under the investments in National Brassica Germplasm Improvement Program project to HR (DAN00117, DAN00208, and DAN1707).

## Conflict of Interest

The authors declare that the research was conducted in the absence of any commercial or financial relationships that could be construed as a potential conflict of interest.
