# Supplementary material for: Genome-Wide Association Mapping Identifies Novel Loci for Quantitative Resistance to Blackleg Disease in Canola
Source: Front Plant Sci. 2020 Aug 11;11:1184. doi: 10.3389/fpls.2020.01184 (PMC7432127; doi:10.3389/fpls.2020.01184)
Supplement: Supplementary file 1 [file Presentation_3.pptx]

## Slide 1
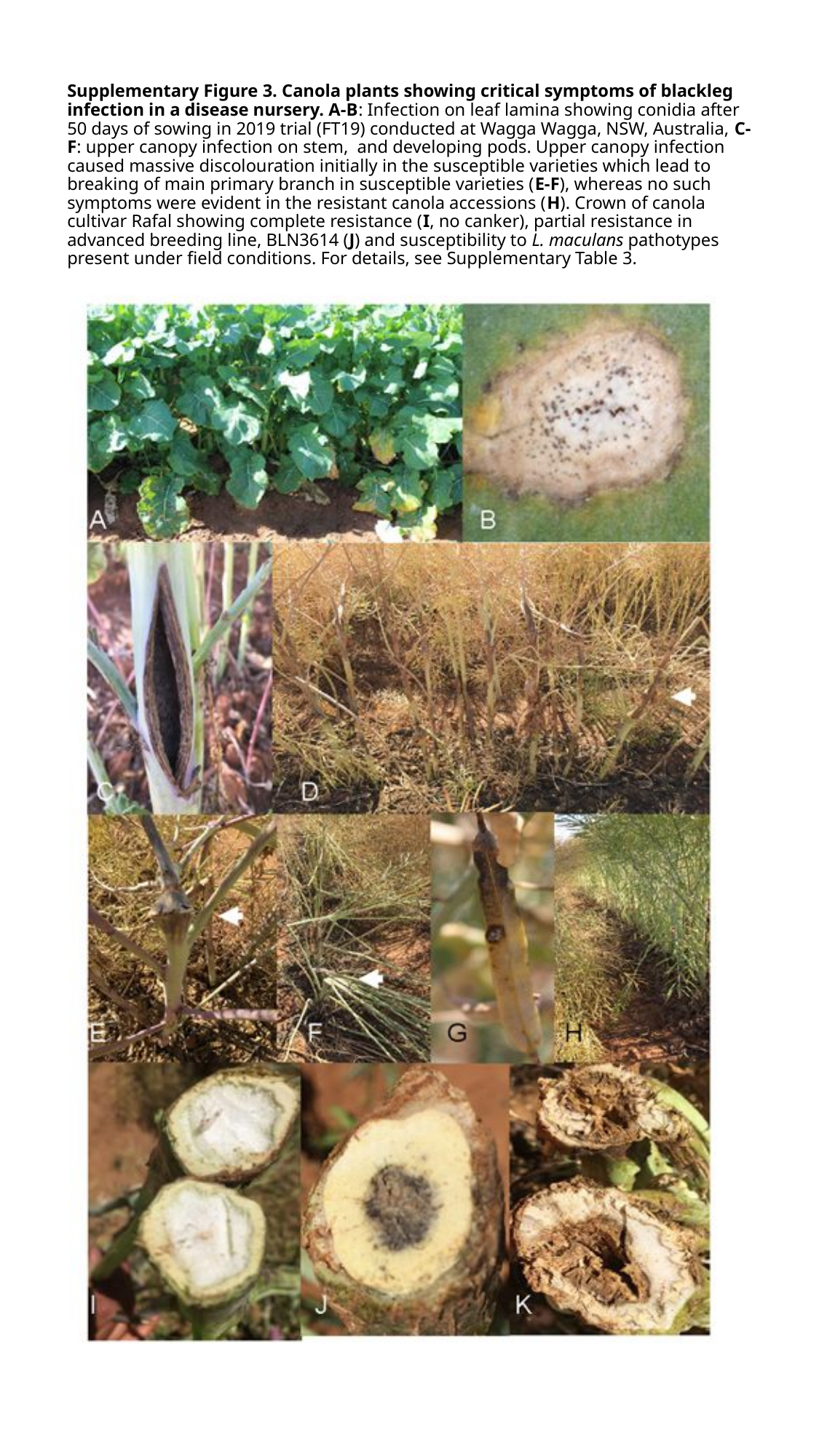

# Supplementary Figure 3. Canola plants showing critical symptoms of blackleg infection in a disease nursery. A-B: Infection on leaf lamina showing conidia after 50 days of sowing in 2019 trial (FT19) conducted at Wagga Wagga, NSW, Australia, C-F: upper canopy infection on stem, and developing pods. Upper canopy infection caused massive discolouration initially in the susceptible varieties which lead to breaking of main primary branch in susceptible varieties (E-F), whereas no such symptoms were evident in the resistant canola accessions (H). Crown of canola cultivar Rafal showing complete resistance (I, no canker), partial resistance in advanced breeding line, BLN3614 (J) and susceptibility to L. maculans pathotypes present under field conditions. For details, see Supplementary Table 3.
