# Supplementary material for: Genome-Wide Association Mapping Identifies Novel Loci for Quantitative Resistance to Blackleg Disease in Canola
Source: Front Plant Sci. 2020 Aug 11;11:1184. doi: 10.3389/fpls.2020.01184 (PMC7432127; doi:10.3389/fpls.2020.01184)
Supplement: Supplementary file 6 [file DataSheet_4.docx]

Supplementary Table 4: Summary of the Illumina SNP markers mapped in a diverse panel of accessions, including the number of markers, length and average spacing between pairs of markers for each linkage group. The distances are provided in kb.

| Linkage group | Number of markers | Length (kb) | Average spacing between markers (kb) |
| --- | --- | --- | --- |
| A01 | 540 | 22,788.26 | 7,434.69 |
| A02 | 492 | 24,726.78 | 8,744.15 |
| A03 | 949 | 29,097.65 | 9,534.34 |
| A04 | 613 | 19,071.06 | 7,359.81 |
| A05 | 592 | 22,643.78 | 8,306.27 |
| A06 | 673 | 24,367.38 | 9,357.78 |
| A07 | 857 | 23,873.45 | 8,132.36 |
| A08 | 466 | 18,874.62 | 6,524.16 |
| A09 | 428 | 33,484.40 | 12,759.90 |
| A10 | 549 | 17,219.63 | 6,110.21 |
| Ann | 321 | 48,527.45 | 15,818.32 |
| **An subgenome** | **6480** | **284674.46** | **9,098.36** |
| C01 | 586 | 38,053.94 | 12,823.81 |
| C02 | 486 | 45,370.16 | 17,156.27 |
| C03 | 1,050 | 60,543.10 | 21,282.56 |
| C04 | 884 | 48,773.15 | 18,661.83 |
| C05 | 414 | 42,868.44 | 16,603.60 |
| C06 | 620 | 36,905.01 | 13,919.98 |
| C07 | 671 | 44,230.84 | 14,075.97 |
| C08 | 561 | 38,243.17 | 11,901.58 |
| C09 | 263 | 48,129.15 | 17,853.86 |
| Cnn | 399 | 80,303.19 | 24,119.48 |
| **Cn subgenome** | **5934** | **483420.15** | **16,839.89** |
| **Total AnCn genome** | **12414** | **768,094.61** | **12,784.81** |

|  |  |  |
| --- | --- | --- |
