# Supplementary material for: Genome-Wide Association Mapping Identifies Novel Loci for Quantitative Resistance to Blackleg Disease in Canola
Source: Front Plant Sci. 2020 Aug 11;11:1184. doi: 10.3389/fpls.2020.01184 (PMC7432127; doi:10.3389/fpls.2020.01184)

**Supplementary Figure 6: Manhattan plots showing genomic regions associated with the phenological traits in a diverse panel of canola.** Flowering time (A), plant height (B) and maturity (C) were evaluated across 2-3 field environments at Wagga Wagga. LOD scores (-log_10_*P*) for association between Illumina SNP and phenological traits in diverse canola accessions are given on Y axis. LOD scores presented in the Manhattan plots are from Base marker model M1. The red dash line indicates the threshold value for significant SNPs at LOD ≥ 3. Positions (kb) of the highly significant (LOD ≥ 3, P ≤ 0.001) and ‘suggestive’ (LOD < 3, 0.001 < P < 0.05) markers from the final Multi QTL model (M2) are labelled. The physical positions of SNPs are based on the map position on the Darmor-*bzh* genome assembly (Clarke et al., 2016). Details of SNP associations are provided in Supplementary Table 5.


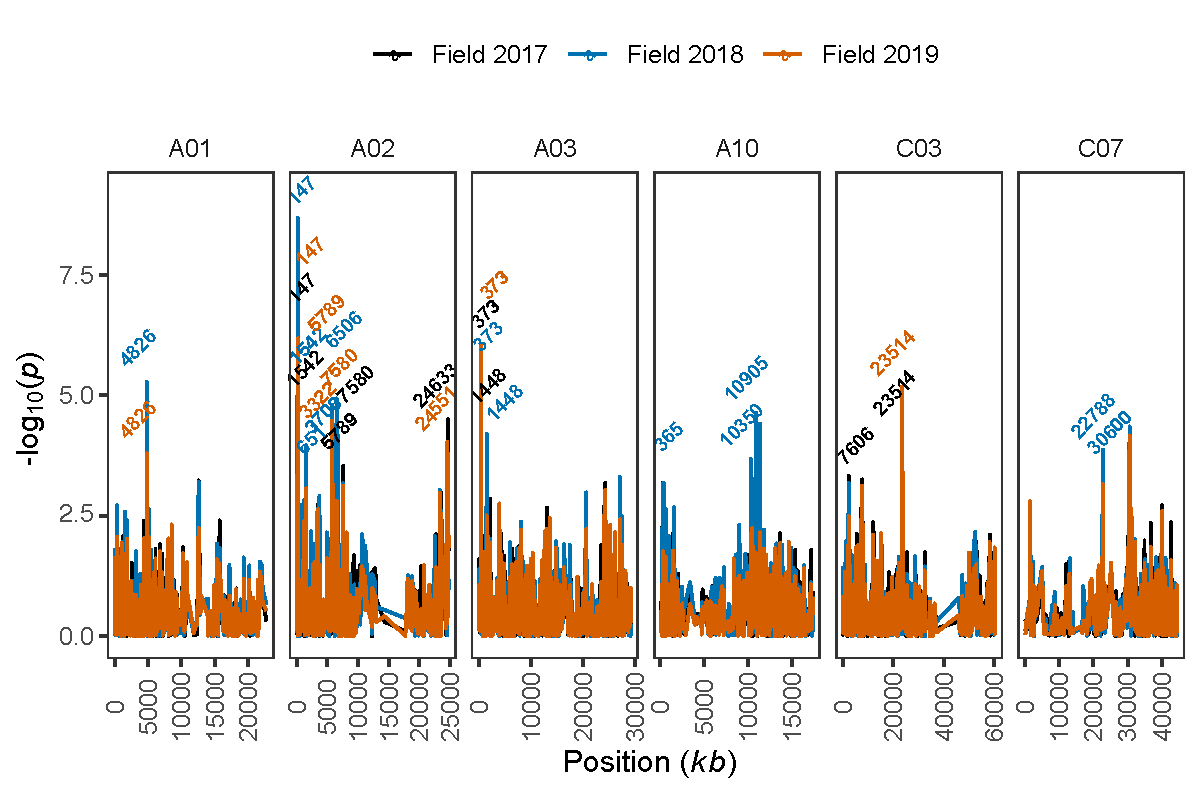


A

B

C


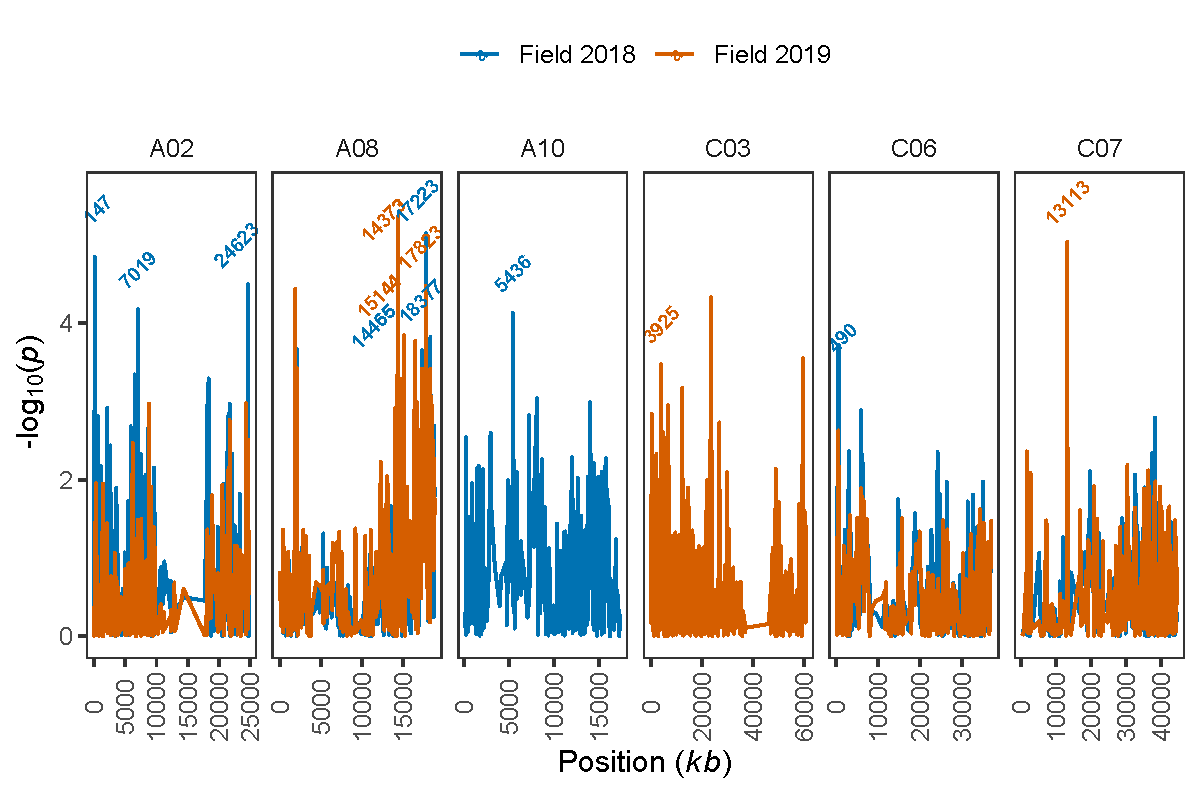


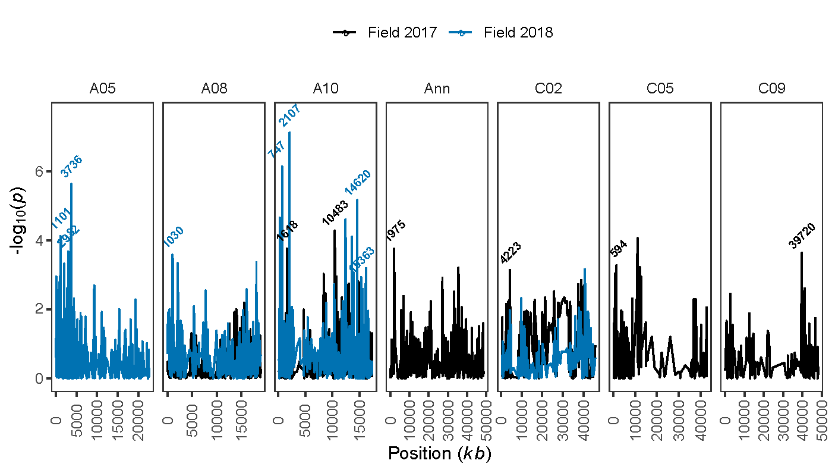

Supplement: Supplementary file 8 [file DataSheet_6.docx]
