# Supplementary material for: Genome-Wide Association Mapping Identifies Novel Loci for Quantitative Resistance to Blackleg Disease in Canola
Source: Front Plant Sci. 2020 Aug 11;11:1184. doi: 10.3389/fpls.2020.01184 (PMC7432127; doi:10.3389/fpls.2020.01184)
Supplement: Supplementary file 10 [file DataSheet_8.docx]

**Supplementary Figure 1:** Lay-out of experiments conducted under shade-house in 2016 (A, SH16), 2017 (B, SH17), and under field conditions in 2017 (FT17) (C). Experiments conducted under field conditions in 2018 (FT18) and 2019 (FT19) followed a similar layout to C, but with a different randomisation of accessions to plots. Details of accessions, evaluated under both field and shade-house environments are given in Supplementary Table 1.

**A**

**B**

**C**
