# Supplementary material for: Genome-Wide Association Mapping Identifies Novel Loci for Quantitative Resistance to Blackleg Disease in Canola
Source: Front Plant Sci. 2020 Aug 11;11:1184. doi: 10.3389/fpls.2020.01184 (PMC7432127; doi:10.3389/fpls.2020.01184)
Supplement: Supplementary file 11 [file DataSheet_9.docx]

**Supplementary Figure 3:** Weather conditions prevailed under field conditions (disease nurseries, Wagga Wagga, NSW 2650, Australia), where evaluation for resistance to blackleg occurred during three canola growing seasons in 2017, 2018 and 2019.
