# Supplementary material for: Genome-Wide Association Mapping Identifies Novel Loci for Quantitative Resistance to Blackleg Disease in Canola
Source: Front Plant Sci. 2020 Aug 11;11:1184. doi: 10.3389/fpls.2020.01184 (PMC7432127; doi:10.3389/fpls.2020.01184)

Supplementary Figure 4: Linkage disequilibrium (LD) across different linkage groups of *B. napus.* LD was evaluated by calculating the *r*^2^ coefficient using 12414 SNP markers.


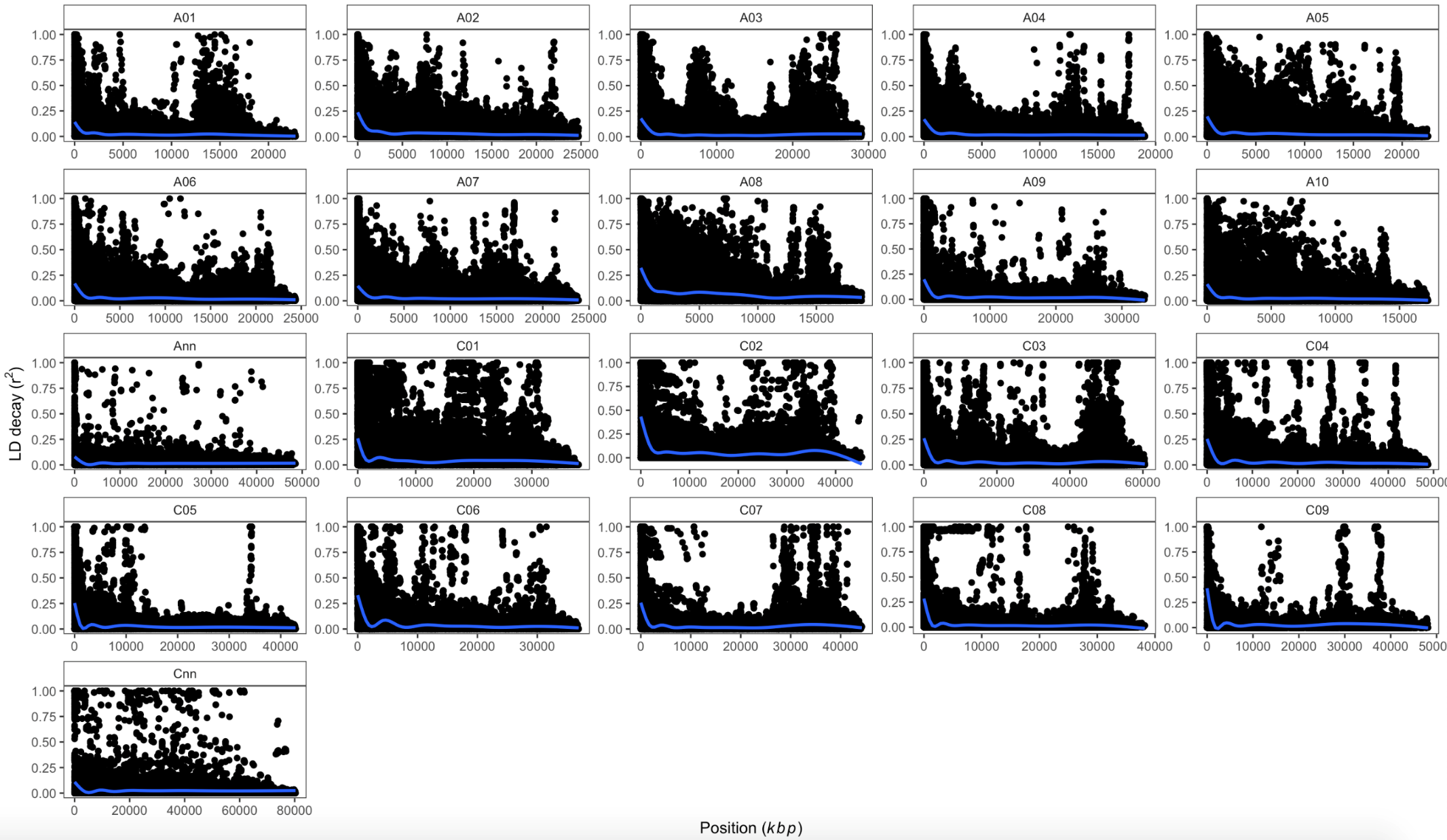

Supplement: Supplementary file 12 [file DataSheet_10.docx]
