# Supplementary material for: Genome-Wide Association Mapping Identifies Novel Loci for Quantitative Resistance to Blackleg Disease in Canola
Source: Front Plant Sci. 2020 Aug 11;11:1184. doi: 10.3389/fpls.2020.01184 (PMC7432127; doi:10.3389/fpls.2020.01184)
Supplement: Supplementary file 13 [file DataSheet_11.docx]

Supplementary Figure 5: Distribution of significant SNP associations for resistance to blackleg, identified across environments on different linkage groups of *B. napus*.
